# Dengue outbreaks in China during 2005-2023: a systematic analysis

**DOI:** 10.3389/fcimb.2026.1920586

**Published:** 2026-08-18

**Authors:** Kun Peng, Xiaodan Huang, Pengxiang Huang, Hongli Zhao, Xiao Pan, Nuo Xu, Hao Yin, Yingchun Yang, Chenxin Han, Maoqing Gong, Hongmei Liu

**Affiliations:** 1 Digestive Disease Hospital of Shandong First Medical University, Jining, Shandong, China; 2 Shandong Institute of Parasitic Diseases, Shandong First Medical University & Shandong Academy of Medical Sciences, Jining, Shandong, China; 3 Shandong Center for Disease Control and Prevention, Jinan, Shandong, China

**Keywords:** China, clinical features, dengue, epidemiology, spatiotemporal distribution

## Abstract

**Objectives:**

Dengue fever is a major global public health concern. While China has not yet established stable, local transmission of the disease, imported cases frequently lead to local dengue epidemics throughout the year. This paper aims to outline the characteristics of dengue outbreaks in China between 2005 and 2023.

**Methods:**

We collected literature on dengue in China from various databases and extracted relevant data for analysis. This data included patients’ sociodemographic characteristics, serotype composition, and clinical manifestations, which we then summarized and compared, evaluating the relationship between dengue virus (DENV) serotypes and clinical manifestations, and analyzing the monthly and regional distribution patterns of dengue fever cases based on case data obtained from the China Public Science and Health Data Center (CPC).

**Results:**

In the included studies, the ratio of male to female was approximately 1:1, The age group with the highest proportion of dengue patients is those aged 30 to 39. Their occupations were predominantly business service, and the predominant serotype of the infection was DENV-1, followed by DENV-2. The major clinical signs and symptoms in dengue patients were fever, asthenia, and headache. Patients infected with DENV-1, 3 were associated with some specific clinical phenotypes. Dengue cases typically peak between September and October each year. Although these cases are primarily concentrated in the southern regions of China, there is a noticeable trend of increasing incidence in northern areas over time.

**Conclusions:**

This study offers an in-depth examination of the molecular epidemiology, clinical characteristics, and the seasonal and geographic distribution of dengue cases in China from 2005 to 2023. The findings provide valuable insights for future research on dengue epidemics.

## Introduction

The DENV is the pathogen responsible for dengue, a disease transmitted by arthropods. DENV is classified as a single-stranded positive-sense RNA virus and falls under the *Orthoflavivirus* genus within the *Flaviviridae* family (Roy and Bhattacharjee, 2021; Osawa et al., 2023; Zhang et al., 2023). DENV is categorized into four distinct serotypes (DENV-1-4), each defined by the unique antigenic properties of its envelope protein E. Infections caused by different serotypes of the DENV can result in more severe clinical outcomes, primarily due to a phenomenon known as antibody-dependent enhancement(ADE) (Pham et al., 2024; Ying et al., 2024). The human body acquires long-lasting specific immunity to a particular DENV serotype following infection, while cross-protection against other serotypes tends to be more short-lived (Montoya et al., 2013; Reich et al., 2013). The clinical manifestations of the different serotypes exhibit slight variations, especially concerning laboratory indicators, clinical symptoms, and the severity of the disease. Between 2000 and 2019, the reported cases of dengue surged from 500,000 to 5.2 million worldwide, leading the WHO to classify dengue as one of the top ten global health challenges in 2019.

The prevalence of dengue fever in China is largely attributable to imported cases, with instances originating in Southeast Asian countries often precipitating localized outbreaks. Since the initial outbreak in Foshan City, Guangdong Province, in 1978, China has witnessed sporadic cases of dengue (Liu, 2019). This disease was officially classified as a legally designated infectious disease in the 1990s (Xiong and Chen, 2014). These cases occurred between June and November, typically peaking in September or October, and are largely confined to southern coastal regions and adjacent provinces, including Hainan, Yunnan, Guangxi, Guangdong, and Fujian (Yan-ding et al., 2022; Yue et al., 2023). Recent studies suggest that dengue cases had spread northward in China as a result of global warming and other factors. In 2017, Jiaxiang County, Jining City(34°25' ~35°55'N), Shandong Province reported cases of dengue (Liu et al., 2020). In 2019, Puyang City (35°20'~36°12'N), Henan Province also reported an outbreak of locally transmitted DENV, marking the northernmost region in China where locally transmitted DENV has been recorded to date (Xu et al., 2021). The growing frequency of cultural and economic exchanges between China and the global community has resulted in a higher number of imported cases and a significant rise in local outbreaks of dengue (Di et al., 2017; Araf et al., 2024). The number of regions experiencing dengue outbreaks in China is increasing, making it a social and public health issue that requires urgent attention. The creation of effective vaccines involves navigating several challenges, including the need to address multiple serotypes and the risks associated with ADE. Several dengue vaccines have now been approved for use, of which CYD-TDV and TAK-003 are the most widely used and have the longest track record of use (Godói et al., 2017; Malisheni et al., 2017; Huang et al., 2021; Sáez-Llorens et al., 2025). While CYD-TDV offers safe and effective protection for individuals previously infected with DENV (seropositive individuals), it elevates the risk of severe disease in those experiencing their first natural DENV infection (seronegative individuals). As a result, pre-vaccination screening strategies have been suggested to identify prior infection history. However, these additional screening requirements have significantly restricted the vaccine’s implementation within national immunization programs (World Health Organization, 2024). Although TAK-003 is generally effective, there are still gaps in the data regarding its protective efficacy against DENV-3 and DENV-4 in individuals who were seronegative at baseline; the World Health Organization’s SAGE has noted that the risk of severe disease following infection with these two serotypes in such individuals cannot be entirely ruled out; Furthermore, the two-dose schedule (at months 0 and 3) may present challenges in terms of adherence; these factors collectively limit its roll-out in large-scale vaccination programs (Escudero et al., 2026). As the world’s second most populous nation, China is predominantly characterized by subtropical and temperate climates, which, combined with its geographic proximity to Southeast Asia, renders it particularly susceptible to both imported and domestic outbreaks of dengue. Therefore, a comprehensive understanding of the evolution of dengue in the country is essential. Currently, prevention and control efforts primarily emphasize preventive strategies, while detailed analyses of the clinical characteristics of dengue patients and the relationships among DENV serotypes in China remain limited. Furthermore, the spatial distribution patterns of dengue outbreaks across the country are not well-defined. This paper systematically reviews dengue cases in China from 2005 to 2023 to clarify their epidemiological characteristics, such as patients’ sociodemographic attributes and clinical manifestations. Correlation analyses between DENV serotypes and clinical manifestations are conducted, alongside descriptions of the monthly distribution of dengue cases. We also collected dengue fever case data from all Chinese provinces and municipalities between 2006 and 2020. By visualizing this data, we identified the spatial distribution patterns of dengue fever cases in China. These insights can improve prevention and control strategies to reduce the spread of outbreaks.

## Methods

### Searching strategy and selection criteria

**Figure 1:**
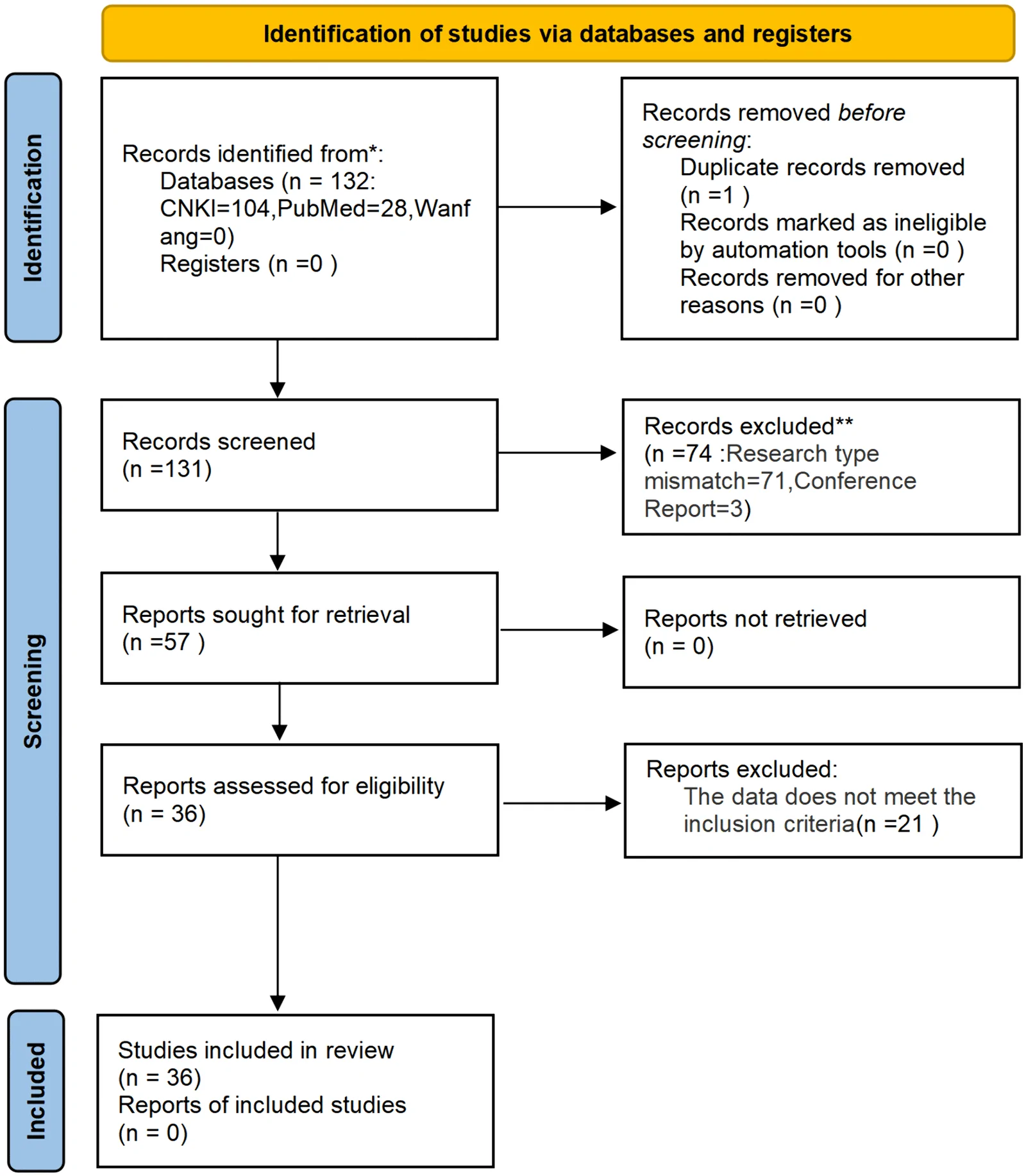
Literature screening flowchart. Rules and processes for searching, screening, and inclusion of literature. *: Consider, if feasible to do so, reporting the number of records identifed from each database or register searched (rather than the total number across all databases/registers). **: If automation tools were used, indicate how many records were excluded by a human and how many were excluded by automation tools.

This study was designed as a systematic review of the literature, complemented by a secondary analysis of publicly available monthly dengue surveillance data to describe spatiotemporal patterns where published data were incomplete. We collected literature published between 2005 and 2024 from the CNKI, Wanfang and PubMed databases, reporting on dengue fever cases that occurred between 2005 and 2023 (**Figure 1**). Our search utilized the following query: CNKI, Wanfang, subject terms: SU = (‘dengue (denv)’ + ‘dengue virus (denv)’ + ‘denv + denv virus’) * (‘China’) * (epidemiology’ + ‘epidemiological characteristics’ + ‘outbreak’ + ‘epidemic’ + ‘clinical characteristics’). Date range: From January 1, 2005 to January 1, 2024. PubMed, (“Dengue”[Mesh] OR “Dengue Virus”[Mesh] OR “dengue”[Title/Abstract] OR “dengue fever”[Title/Abstract] OR “dengue virus”[Title/Abstract] OR “DENV”[Title/Abstract]) AND (“China”[Mesh] OR “China”[Title/Abstract] OR “Chinese”[Title/Abstract]) AND (“epidemiology”[Subheading] OR “Epidemiology”[Mesh] OR “epidemiology”[Title/Abstract] OR “clinical characteristic*”[Title/Abstract]), Period: 2005–2024. The most recent retrieval date was April 7, 2025. Three authors conducted the screening independently, without the use of automated tools, and consolidated the results as follows: abstracts were reviewed to eliminate articles that did not focus on dengue cases. In cases where multiple articles reported on the same dengue outbreak, all pertinent literature was included, and non-duplicated data were extracted. Additional selection criteria mandated that articles must present data on dengue cases in China from 2005 to 2023, including details such as DENV serotype and clinical characteristics of patients. For data utilization, literature from studies within the same category was grouped together to facilitate comprehensive data analysis. The critical appraisal tool for prevalence studies designed by the Joanna Briggs Institute (JBI) was used to assess the risk of bias in all included studies (**Supplementary Table S1**), The findings reveal that the studies analyzed exhibited a high level of overall reliability; however, certain studies presented a potential risk of confounding bias.

### Data extraction and analysis

We obtained the full texts of the studies that met our inclusion criteria and extracted relevant data, including authors, publication year, city, patient demographics, clinical symptoms, and DENV serotypes. We used study size and case numbers to characterize epidemiology, clinical presentation, and serotype distribution among the different study groups. Due to the varying levels of detail in the included literature regarding study indicators, we aimed to group studies with similar classification criteria and indicators for data selection. As a result, the number of studies included in each category varies (**Supplementary Table S2** for details on inclusion). After recording all data in Excel spreadsheets, we utilized various software packages for analysis. A heatmap was created to illustrate the distribution of occupational and age characteristics among dengue patients. Tables documented the probabilities of various clinical symptoms occurring in these patients. We generated graphs using Stata MP-64 software to depict the relationship between the three DENV serotypes and the patients’ clinical manifestations. Since the monthly and provincial dengue case data extracted from the literature were incomplete and discontinuous, we supplemented the data with case records obtained from the CPC to better characterize the monthly and spatial distribution patterns of dengue cases. These data were used exclusively for the analysis of monthly and spatial distributions. Although our study period covered 2005-2023, monthly case data were only available for 2006-2020 due to data access restrictions. SPSS was used to analyze variations in the monthly distribution of dengue cases, and graphical representations were created in Prism. Additionally, the distribution of DENV serotypes across different cities and the northward trend map of dengue cases in China were generated using ArcMap software (version 10.8.2). This study adheres to the Preferred Reporting Items for Systematic Reviews and Meta-Analyses (PRISMA) checklist, as detailed in the **Supplementary Materials** (**Supplementary Table S6**, **S7**).

### Statistical methods

The study examined various factors related to dengue patients, including occupational composition, age distribution, Serotype composition of DENV serotypes, and clinical characteristics. To analyze this data, we applied the Freeman–Tukey double inverse sine transformation in R Studio to pool data of the same type from multiple studies, including the male-to-female ratio, age distribution, occupational distribution, prevalence of dengue serotypes and clinical characteristics of dengue patients. A random-effects model was used when *I*² > 50%; otherwise, a fixed-effects model was employed. We used the chi-squared goodness-of-fit test to analyze differences in the occupational distribution and age distribution of dengue patients, as well as the prevalence of dengue serotypes. For analyzing variations in the monthly distribution of dengue cases, we employed a non-parametric approach, specifically the Kruskal-Wallis test. When assessing heterogeneity, we employed two methods—the DerSimonian-Laird (DL) method and the Restricted Maximum Likelihood (REML) method—depending on the data differences; the DL method was used when heterogeneity was high, and the REML method in all other cases. Unless otherwise stated in the figure captions, the REML method was used by default to assess heterogeneity.

## Results

### Systematic review

Three members of the research team identified 132 articles through electronic literature searches, 36 of which were utilized for our analysis. A summary of the data from the investigations included is presented in **Figure 1**. These studies examined the dengue outbreak across seven Chinese provinces and autonomous regions. The locations and the number of studies conducted in each area are as follows: Jiangxi Province (n=1), Fujian Province (n=2), Guangdong Province (n=18), Yunnan Province (n=9), Zhejiang Province (n=3), Hunan Province (n=1), and Guangxi Zhuang Autonomous Region (n=1).

### Social attributes of dengue patients

**Figure 2:**
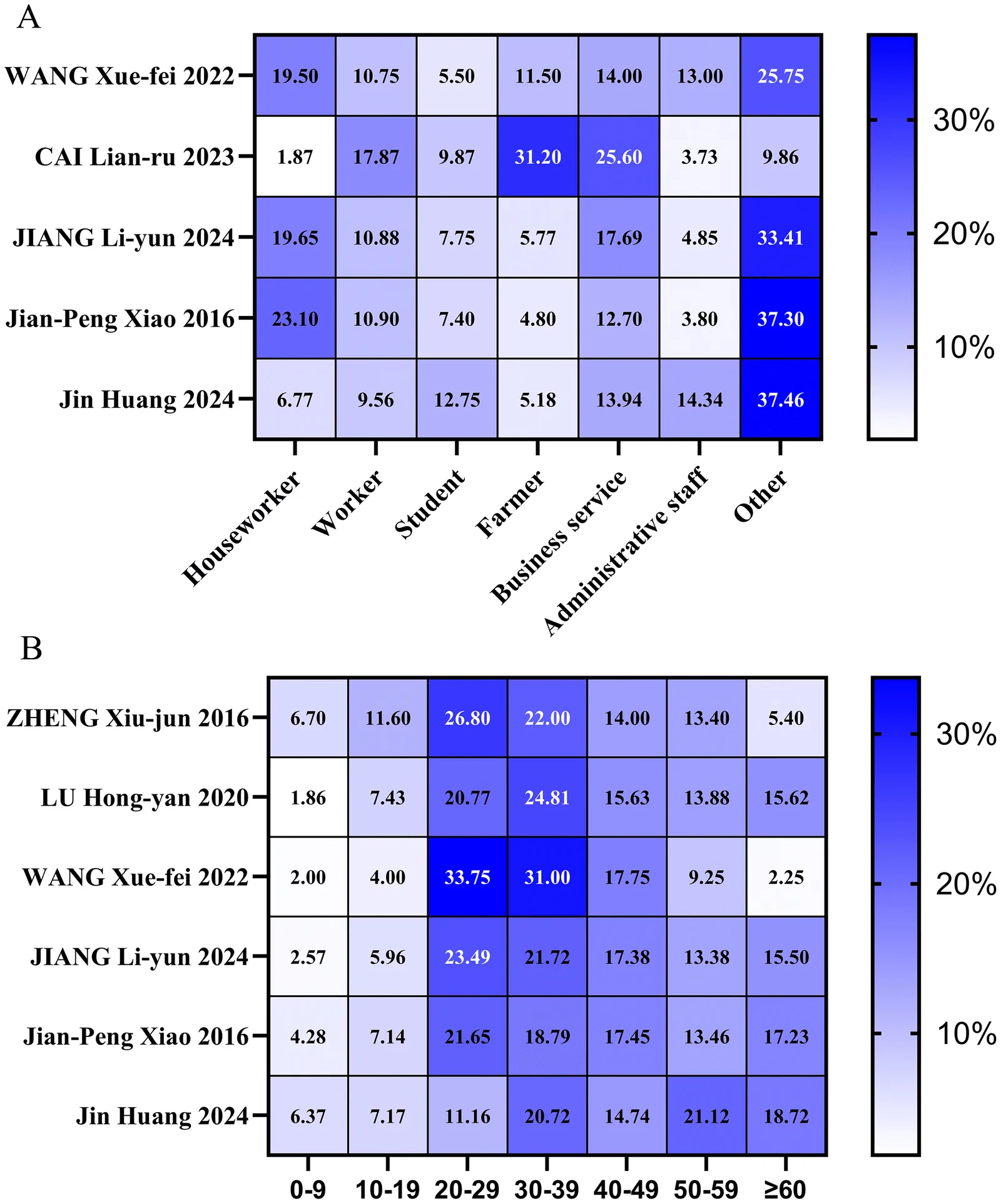
Heat map of occupational composition and age distribution of dengue patients. **(A)** illustrates the occupational distribution of dengue patients derived from five studies. The horizontal axis categorizes occupations as defined in the referenced literature, while the vertical axis reflects the name of studies included in the analysis. **(B)** presents the age distribution of dengue patients based on data from six studies. The horizontal axis categorizes age groups according to the classification criteria outlined in the referenced literature, whereas the vertical axis shows the name of studies contributing to this data.

Regarding occupational composition, business service accounted for the highest proportion at 16.53% (95% CI: 8.51–26.57%), followed by houseworker at 12.88% (95% CI: 0.45–38.29%), followed by worker at 11.81% (95% CI: 7.49–16.97%). The lowest proportion was administrative staff at 7.25% (95% CI: 0.82–19.28%) (*df*=6, *χ²*=17.565, *P*< 0.01). Concerning age distribution, based on the age grouping employed in the majority of included studies, patients were categorized into seven decadal age groups. The small number of participants aged 60 years and above was collectively assigned to the ≥60 age group. The 30–39 age group exhibited the highest proportion at 22.98% (95% CI: 15.89–30.94%), followed by those aged 20–29 at 22.61% (95% CI: 10.43–37.81%). The lowest proportion was observed among those aged 0–9, at 3.59% (95% CI: 1.01–7.67%) (*df*=6, *χ*²=22.640, *P*< 0.001)(see **Figure 2** for details).

### Serotype distribution of DENV in China

**Figure 3:**
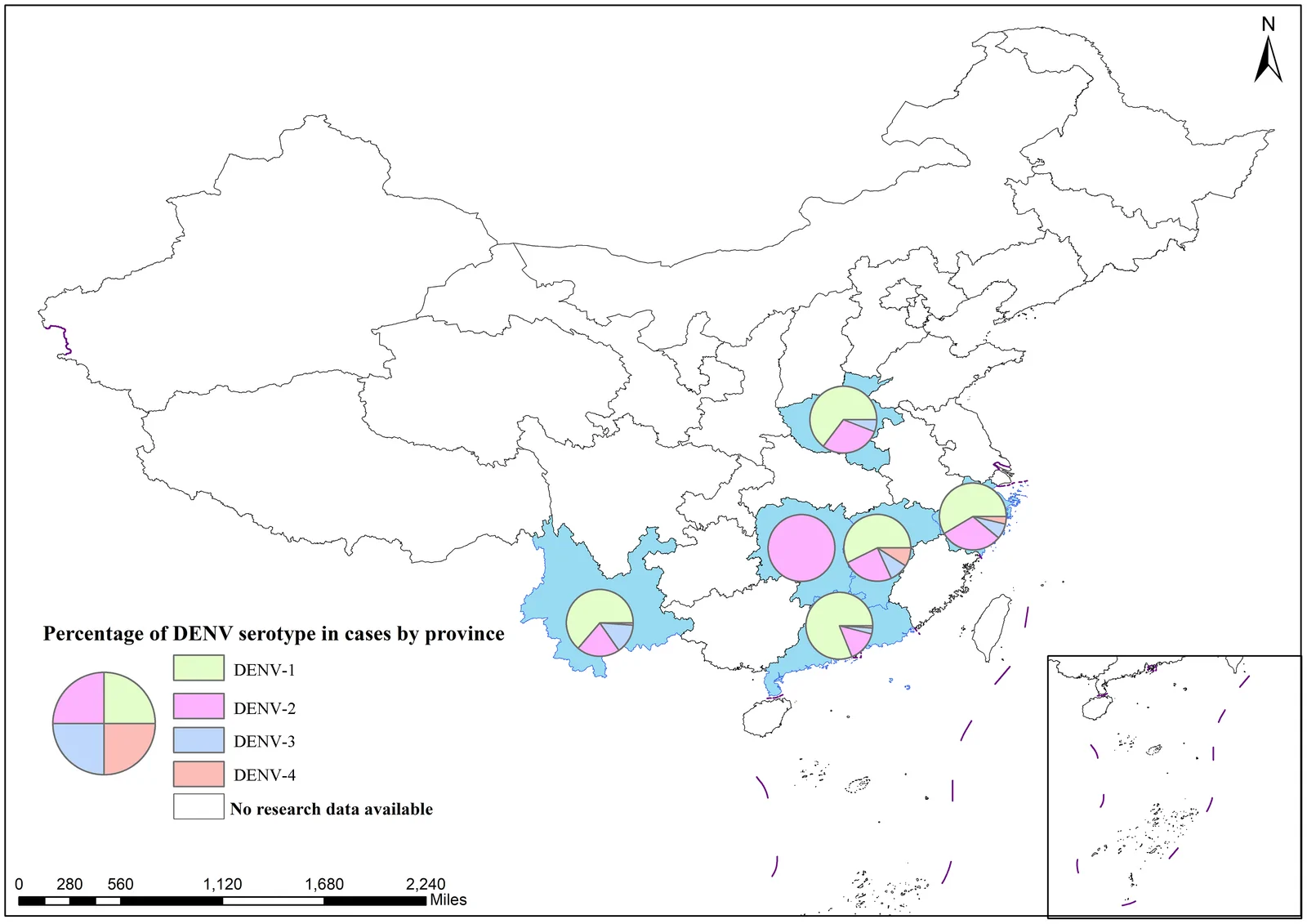
Prevalence of DENV serotypes in China. The base map was sourced from the National Platform for Common Geospatial Information Services (Tianditu) of the Ministry of Natural Resources of China (https://www.tianditu.gov.cn). Basemap Approval Number: GS(2024)0650.

**Table 1** presents the DENV serotype data extracted from the included studies. We then created visualization maps to illustrate the prevalence of these serotypes. However, data limitations restricted the generation of serotype composition maps to only six provinces (**Figure 3**).

**Table 1:** Prevalence of each DENV serotype in the study.

Serotypes Province	Number of cases	DENV-1(%)	DENV-2(%)	DENV-3(%)	DENV-4(%)	P
Guangdong	4157	80.99	14.70	2.14	0.37	<0.001
Yunnan	1401	61.0	20.1	13.7	1	<0.001
Zhejiang	216	57.5	29.8	7.3	3.6	<0.001
Hunan	96	0	100	0	0	
Jiangxi	375	57.33	24.53	9.07	9.07	<0.001
Henan	17	64.71	29.41	5.88	0	<0.05
All	6262	59.94	28.48	5.52	1.01	<0.001

Among the 15 studies reviewed, we analyzed the serotype composition ratio for each dengue serotype. For the studies conducted in Guangdong and Yunnan, we applied a random-effects model with Freeman-Tukey double arcsine transformation to aggregate the serotype data. In contrast, for the Zhejiang data, which had fewer than three studies available, we utilized a fixed-effects model with the same Freeman-Tukey double arcsine transformation for data pooling.

The proportions for each of the four serotypes were estimated separately using random-effects models. Because each proportion was derived from an independent model, their sum does not necessarily equal 100%. However, the 95% confidence interval for each proportion reflects its associated uncertainty.

The overall proportion of DENV-1 was 59.94%, followed by DENV-2 at 28.48%, while DENV-3 and DENV-4 accounted for only 5.52% and 1.01% respectively (*df*=3, *χ²*=91.147, *P*<0.001). In the examined provinces, DENV-1 emerged as the dominant serotype across all areas, except for Hunan. This anomaly can be attributed to the limited number of literature sources available for Hunan. The next most common serotypes were DENV-2 and DENV-3. Among the studies that reported molecular tracing results (predominantly based on *E* gene or *C/PrM* gene sequencing and phylogenetic genotyping), all DENV serotypes examined showed high genetic similarity with previously identified strains from Southeast Asia and India. In the dengue serotype data grouped by year (**Table 2**), DENV-1 remained the dominant serotype for the majority of the period, particularly in 2014 (91.5%), 2017 (87.3%) and 2023 (87.1%), when DENV-1 accounted for over 85% of cases. Between 2015 and 2016, the proportion of DENV-2 rose; in 2015, DENV-2 (45.1%) was almost on a par with DENV-1 (44.2%), with the two serotypes jointly dominating. During the 2020–2021 period, which was characterized by a smaller sample size due to the outbreak, DENV-3 and DENV-4 together accounted for 17.3% (2020) and 20.9% (2021) respectively, which was higher than in most other years, suggesting that sporadic introductions of multiple serotypes occur even during periods of low prevalence.

**Table 2:** Prevalence of dengue serotypes, grouped by year.

Year	Sample size	DENV-1	DENV-2	DENV-3	DENV-4
2014	389	356	26	1	6
2015	113	50	51	8	4
2016	200	120	64	15	1
2017	808	705	78	15	10
2018	1156	808	323	16	9
2019	2657	2018	353	274	12
2020	127	68	37	9	13
2021	158	82	43	17	16
2023	350	305	43	1	1

Data for certain years are missing because the literature included in this study did not contain information on the distribution of dengue serotypes for those years; this does not mean that there were no dengue cases in those years.

### Clinical characteristics of patients with dengue

The clinical manifestations of dengue patients reported in the studies were summarized and categorized. We counted the number of studies included and calculated the incidence rates for each clinical manifestation, along with their corresponding 95% confidence intervals. Fever was the most prevalent clinical symptom (pooled proportion: 99.33% (96.40%, 99.93%)), followed by asthenia (65.43% (33.60%, 91.01%)), headache (60.05%(24.25%, 90.54%)) (*df*=9, *χ²*=178.372, *P*<0. 001). Among laboratory findings, common manifestations included decreased white blood cell (WBC) count (83.02% (74.07%, 90.39%)), decreased platelet (PLT) count (75.60% (62.91%, 86.34%)), elevated Aspartate Aminotransferase (AST) (69.68% (52.07%, 84.72%)) and elevated Alanine Aminotransferase (ALT) (52.98% (32.57%, 72.89%)) (*df*=3, *χ²*=6.993, *P*>0.05) (**Table 3**). This indicates that following DENV infection, patients experience intense immune responses, tissue damage, and hepatocyte injury. In addition, we calculated the proportions of patients with mild and severe dengue and analyzed the data using the Freeman–Tukey double inverse sine transformation. The pooled analysis showed that mild cases accounted for 98.17% (95% CI: 95.42%–99.69%), whilst severe dengue accounted for 1.83% (95% CI: 0.31%–4.58%).

**Table 3:** Characteristics of clinical signs and laboratory investigations in patients with dengue.

Clinical symptoms and signs	No. studies meta-analyzed	Meta-analysis, pooled data (95% CI)
Fever	3	99.33%(96.40%, 99.93%)
Headache	3	60.05%(24.25%, 90.54%)
Rash	3	49.72%(26.84%, 72.67%)
Myalgia	3	53.95%(25.82%, 80.76%)
Asthenia	3	65.43%(33.60%, 91.01%)
Nausea	3	19.57%(9.64%, 31.95%)
Anorexia	3	47.61%(11.31%, 85.48%)
Vomiting	3	14.03%(7.95%, 21.48%)
Diarrhea	3	12.98%(8.38%, 18.41%)
Cough	3	7.06%(3.06%, 12.54%)
Main laboratory results during the course of the disease		
WBC↓(4-10×10^9^)	7	83.02% (74.07%,90.39%)
PLT↓ (100–300× 10^9^)	7	75.60% (62.91%,86.34%)
AST↑	7	69.68% (52.07%,84.72%)
ALT↑	7	52.98% (32.57%,72.89%)

The pooled analysis summarized the clinical manifestations reported across the included studies, indicating both the number of studies contributing data and the proportion of patients exhibiting each symptom. Data were combined using a random-effects model with the Freeman–Tukey double arcsine transformation. WBC↓ denotes leukopenia, PLT↓ denotes thrombocytopenia, AST↑ denotes elevated aspartate aminotransferase, and ALT↑ denotes elevated alanine aminotransferase.

**Figure 4:**
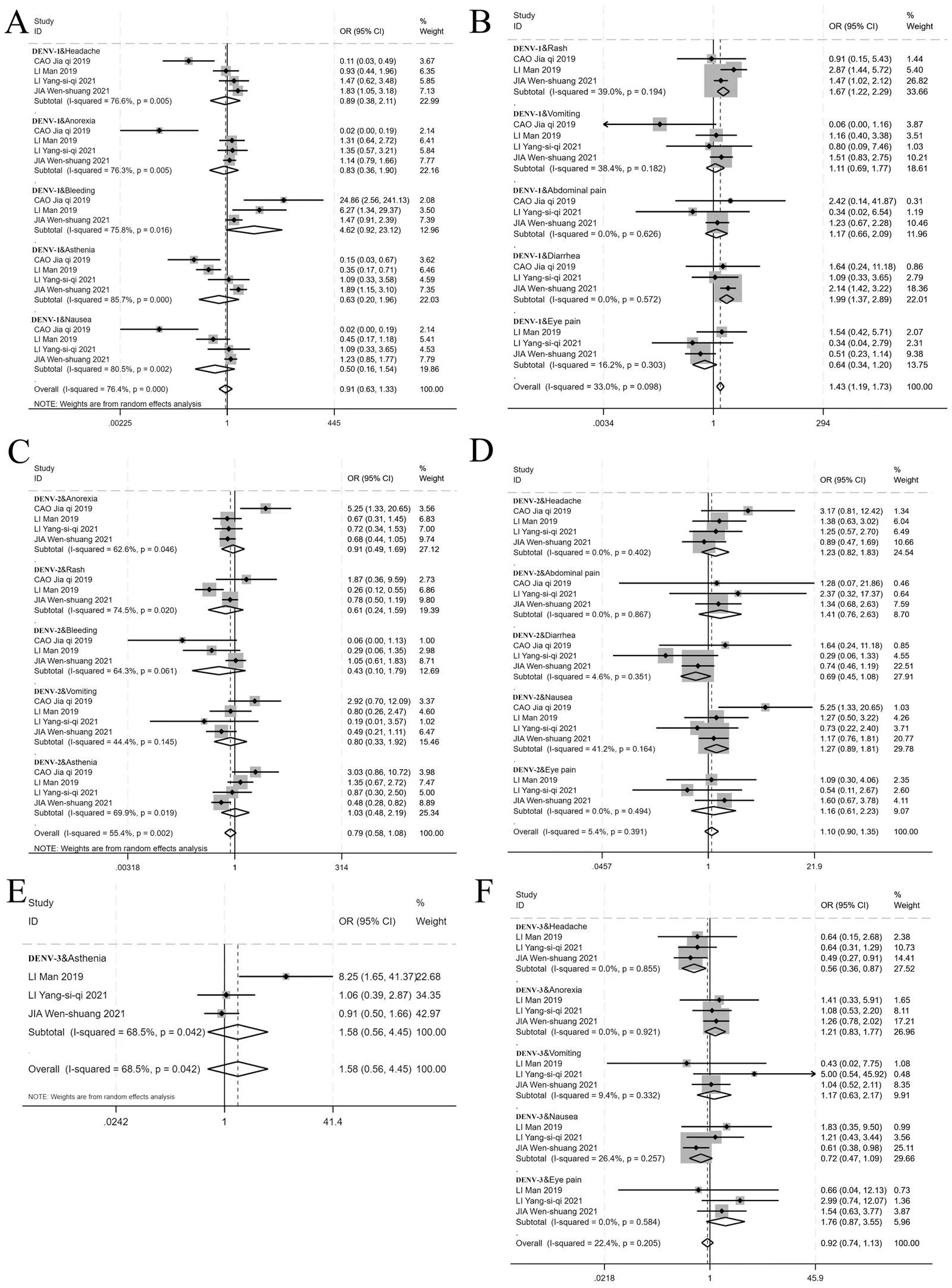
Association of DENV serotypes with clinical characterization of patients. The figure categorizes DENV serotypes into subgroups based on their clinical manifestations. Each row within a subgroup represents an independent study included in the analysis. The ‘Subtotal’ indicates the pooled effect size for that subgroup, with heterogeneity variance estimated using the DL method. The OR and weight ratios for each clinical manifestation associated with the three DENV serotypes are presented. **(A, B)** illustrate the relationship between DENV-1 and various clinical manifestations. **(C, D)** depict the association between DENV-2 and clinical manifestations, while **(E, F)** showcase the connection between DENV-3 and clinical manifestations. For data exhibiting heterogeneity (*I^2^*) of 50% or greater, the I-V heterogeneity model was utilized to calculate odds ratios. Conversely, for data with *I^2^* < 50% utilized the Mantel-Haenszel model was employed. Specifically, **(A, C, E)** utilized the I-V heterogeneity model, while **(B, D, F)** employed the Mantel-Haenszel model.

**Figure 4** presents a forest plot illustrating the association between serotypes and clinical manifestations. An Odds Ratio (OR) confidence interval greater than 1 indicates a positive correlation between the risk factor and the study objective. The size of each block represents the weight, or contribution, of that study within the subgroup, with larger blocks indicating greater weight. Patients with DENV-1 infection showed a statistically significant association with an increased risk of rash (pooled OR = 1.67, 95% CI: 1.22–2.29, *I*² = 39%) and diarrhea (pooled OR = 1.99, 95% CI: 1.37–2.89, *I*² = 0%). In contrast, DENV-3 infection was significantly associated with a lower frequency of headache (pooled OR = 0.56, 95% CI: 0.36–0.87, *I*²= 0%) (see **Figure 4** for details). Patients with DENV-2 were not significantly associated with any specific clinical manifestation (rash, diarrhea, headache or other), as the 95% confidence intervals for all OR included 1.0. This review included five articles reporting the seroprevalence of IgM and IgG antibodies for dengue fever. There was extremely significant heterogeneity between the studies (IgM: *I*² = 99.5%, Q = 863.04, *P* < 0.001; IgG: *I*² = 98.6%, Q = 289.73, *P* < 0.001). In four of these studies, the IgG positivity rate was below 25%, with two studies reporting rates below 10%. These findings suggest that in certain regions or among populations surveyed recently, the prevalence of prior dengue infection is low and the herd immunity barrier is weak; consequently, the proportion of susceptible individuals may be high in the event of an outbreak. However, a 2019 study showed an IgG positivity rate as high as 76.79%, indicating that, in specific populations (such as those in areas of long-term endemicity or among adults), a significant proportion had already acquired antibodies through natural infection (Cao, 2019). This marked difference is closely related to the study location, the intensity of the outbreak, the year of sampling and the age structure of the population. For example, the IgM positivity rate in CAI’s 2015 study reached as high as 94.61%, suggesting that the study may have coincided with an acute outbreak, at a time when the IgG positivity rate had not yet risen (Cai et al., 2015). Overall, the IgG positivity rates among most of the surveyed populations remain at relatively low levels, falling significantly short of the seropositivity threshold required to achieve herd immunity. This highlights the need to strengthen serological surveillance in priority areas and to identify immunocompromised populations in the absence of effective vaccination.

### Seasonality of dengue cases in China

**Figure 5:**
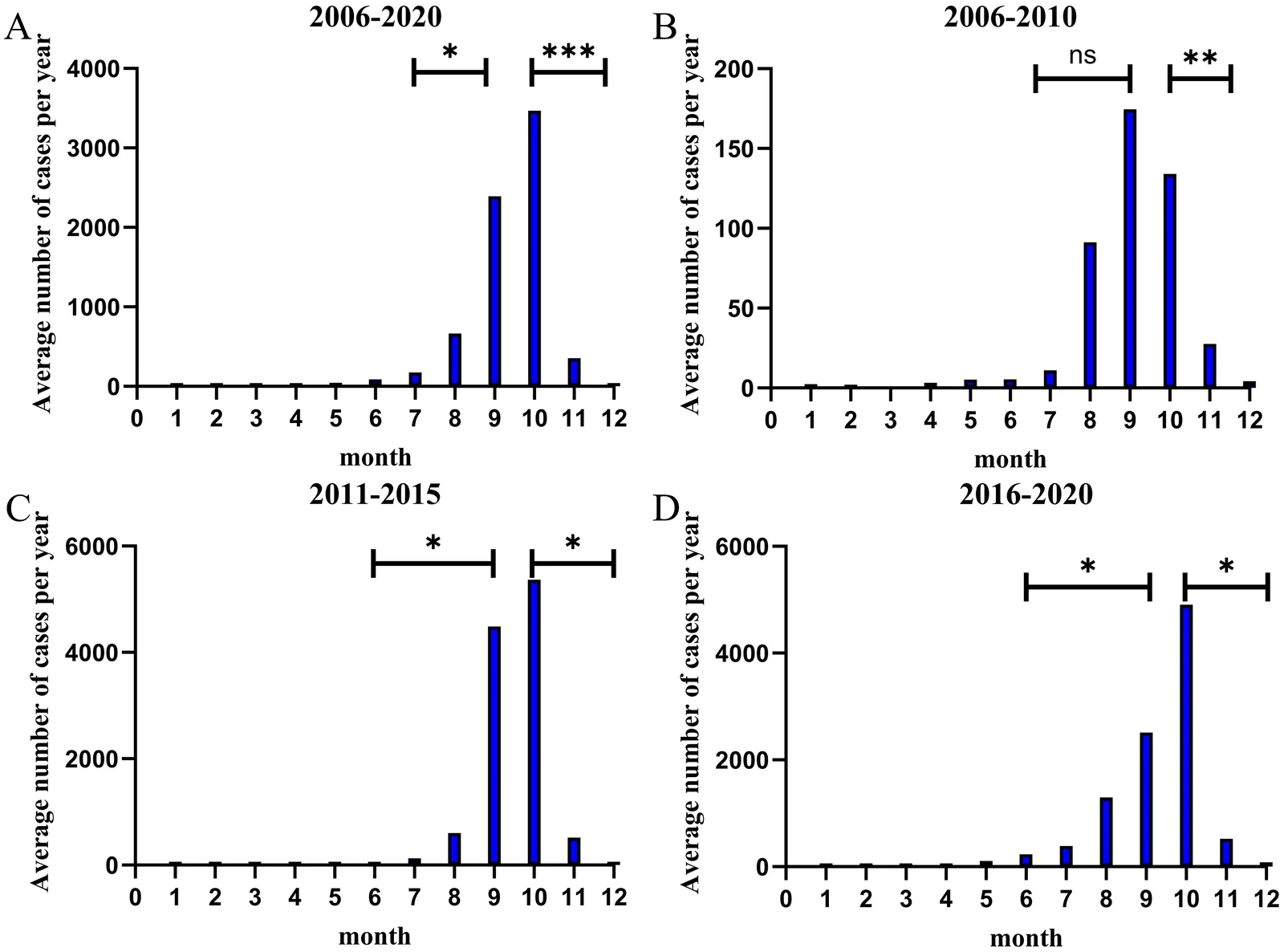
Monthly distribution of dengue cases, 2006-2020. **(A)** is a histogram of the number of cases of dengue by month from 2006 to 2020. **(B)** is a histogram of the number of cases by month for dengue, 2006-2010. **(C)** is a histogram of the number of cases by month for dengue from 2011 to 2015. **(D)** shows the number of cases of dengue by month for the period 2016-2020. “ns” denotes P>0.05, “*” denotes P≤0.05, “**” denotes P≤0.01, “***” denotes P≤0.001.

Monthly dengue case data from 2006 to 2020 were collected from the CPC. Plotting this data as a bar chart reveals significant and statistically significant variations in case numbers between months (*df*=11, *χ²* = 85.909, *p* < 0.001). Dengue cases predominantly occur between August and October, representing about 89.4% of all reported instances (**Figure 5**). The peak in annual cases is observed in September and October, followed by a notable decline in November. Statistical analysis reveals no significant differences in case numbers between September and the preceding months of August or October (*P*=0.516, *P*=0.987). While the average number of cases in October does not significantly differ from that in November (*P*=0.139), it is significantly higher than the case count in December (*P*<0.001). Between 2006 and 2010, domestic dengue cases peaked in September. However, from 2011 to 2015 and then again from 2016 to 2020, the peak gradually shifted to October.

### Spatial distribution of dengue cases in China

**Figure 6:**
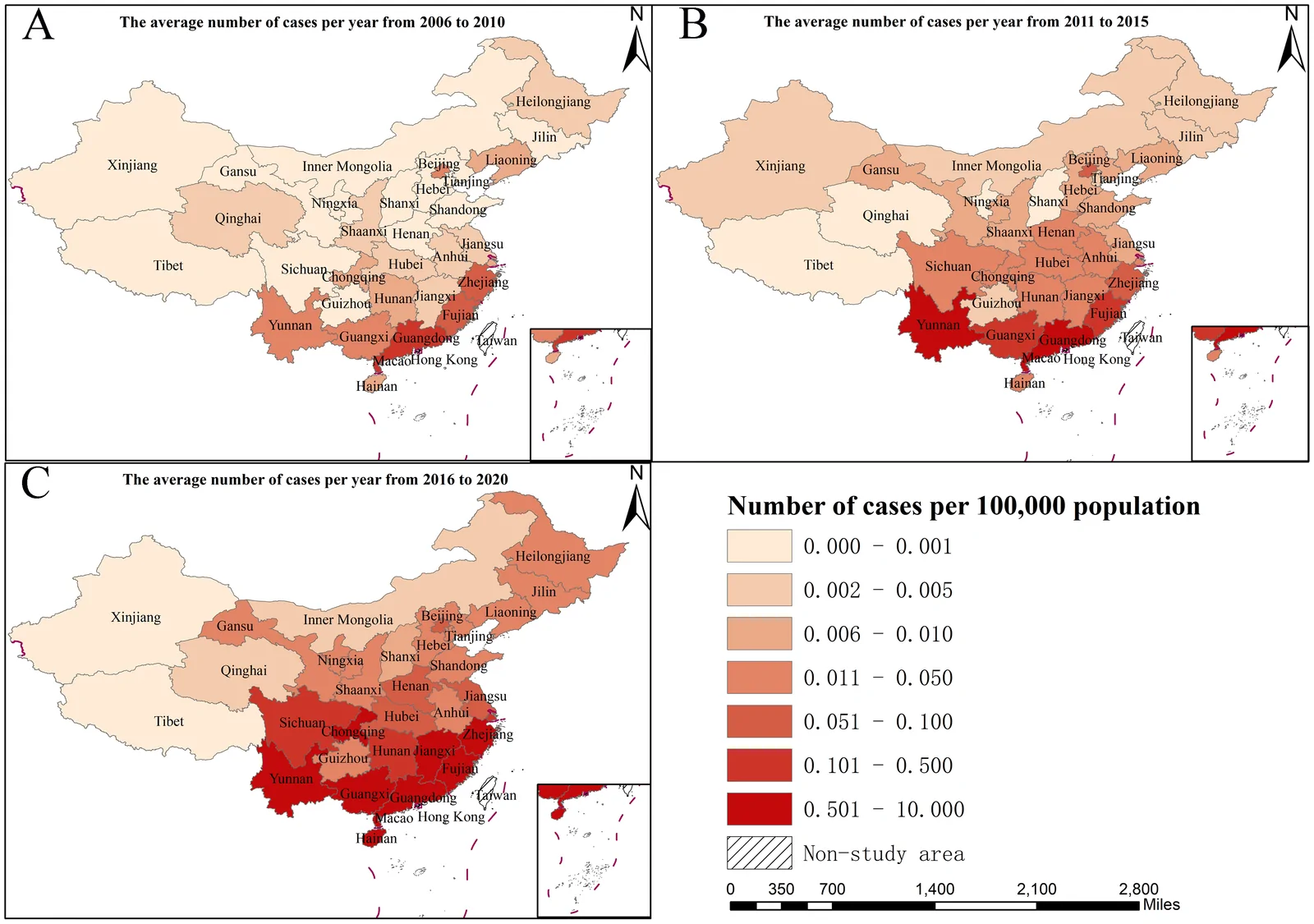
The northward shifting trend of dengue cases from 2006 to 2020. Visualization map of dengue fever cases per 100,000 population by province every five years from 2006 to 2020. **(A)** is for 2006-2010; **(B)** is for 2011-2015; and **(C)** is for 2016-2020. The base map was sourced from the National Platform for Common Geospatial Information Services (Tianditu) of the Ministry of Natural Resources of China (https://www.tianditu.gov.cn). Basemap Approval Number: GS(2024)0650. Population data for each province and autonomous region is sourced from the National Bureau of Statistics.

After examining the literature, we observed that early studies primarily focused on dengue outbreaks in the provinces of Guangdong and Yunnan. However, subsequent research has expanded to include a wider range of provinces and cities, revealing a significant northward shift in the reported geographic distribution of the disease in recent years. To support this finding, we gathered detailed data on dengue cases across all Chinese provinces from the CPC for the period between 2006 and 2020, and obtained historical population data for each province and autonomous region from the National Bureau of Statistics. We created visualization maps that clearly illustrate the increasing number of dengue fever cases moving northward (**Figure 6**). These maps display the number of cases per 100,000 population recorded in each province every five years from 2006 to 2020.

Between 2006 and 2010, dengue cases were primarily concentrated in Guangdong Province, which recorded an average of over 300 cases annually. Meanwhile, Zhejiang, Fujian, and Yunnan Provinces reported sporadic cases, averaging between 20 and 50 each year. In contrast, other provinces experienced negligible instances of the disease. From 2011 to 2015, the cases persisted mainly in Guangdong, Guangxi, and Yunnan Provinces, with reported cases exceeding 100 per year on average. The most severe situation was observed in Guangdong Province, with an average of over 8,000 cases per year. In Sichuan Province, Hunan Province, Hubei Province, Henan Province, and Beijing, the number of cases increased relative to the 2006-2010 period, with an average of over 10 cases per year. During 2016-2020, cases mainly occurred in Zhejiang Province, Fujian Province, Jiangxi Province, Hunan Province, Guangdong Province, Guangxi Province, Chongqing Municipality, and Yunnan Province. Henan Province, Hainan Province, and Sichuan Province also saw an average of nearly 100 dengue cases per year. Notably, in 2017, Jiaxiang County in Jining City, Shandong Province, reported 79 indigenous cases. This was the first year in which Shandong Province recorded over 100 dengue cases. Two years later, in 2019, Puyang City in Henan Province reported 64 indigenous cases, representing the northernmost location in China to have reported such cases at that time. In the same year, Henan Province recorded its highest annual number to date, with 285 cases.

Collectively, these surveillance data indicate a geographic expansion of reported dengue cases toward higher-latitude provinces in China over the study period.

## Discussion

From 2005 to 2023, we conducted a study on dengue in China that examined: (1) the clinical characteristics of dengue patients and their relationship with DENV serotypes; (2) the monthly and spatial distribution trends of dengue cases; and (3) the overall epidemiology of the disease. The provinces of Guangdong, Yunnan, and Fujian reported the highest number of dengue cases. These areas, with their tropical and subtropical climates, create optimal conditions for the proliferation of DENV vectors, supported by favorable temperatures and rainfall patterns. Furthermore, their geographical closeness to Southeast Asia, combined with their roles as major tourist and commercial hubs in China, renders them particularly susceptible to imported cases from abroad, which heightens the risk of domestic outbreaks due to increased travel. Notably, Wang et al. reported that the DENV affecting the Xishuangbanna and Dehong districts in 2013 originated from Southeast Asian countries. On the other hand, Song et al. examined the genotypes of viruses found in Guangdong Province in 2013 and discovered that the strains were initially introduced from Thailand to Guangzhou City, after which they spread to Foshan and Zhongshan, and subsequently from Zhongshan to Jiangmen (Sang et al., 2016; Wang et al., 2016). Southeast Asia and other regions face significant socioeconomic challenges that hinder the full implementation of dengue prevention and control measures. As a result, recent years have seen increasingly severe dengue outbreaks, raising the risk of epidemic situations. This has intensified the efforts required to prevent the importation of the DENV into China’s border cities. To address these challenges and reduce the likelihood of future epidemics spreading to other areas, Guo et al. have proposed that border cities develop robust laboratory disease surveillance systems and implement coordinated vector management strategies (Guo et al., 2015). Research on the sociodemographic characteristics of dengue patients reveals that the most affected occupational groups are commercial service workers, domestic workers, and the unemployed, with manual laborers following closely. Our research shows that the 30–39 age group has the highest percentage of dengue cases, a finding that merits further investigation alongside seroepidemiological evidence. For example, in a study using serological data and modeling to estimate DENV transmission intensity in China, researchers found age to be a significant factor, with the elderly or adolescents identified as high-risk groups (Li et al., 2023). The age distribution in disease reports may not accurately reflect the age-specific risk of infection. Several factors could explain this discrepancy. First, adults aged 30–39 may be more likely to seek medical care or be aware of clinical symptoms, resulting in increased reporting. Second, this cohort may have different histories of childhood exposure compared to younger or older generations, potentially representing a group with a higher proportion of primary infections. Third, studies show that human movement for work and leisure significantly influences DENV transmission at finer spatial scales, such as regional, intra-urban, and neighborhood levels (Gouveia et al., 2025). Occupational activities and migration patterns may increase exposure risk to vector habitats in this age group, which also constitutes a high proportion of the overall population. We acknowledge that, lacking seroepidemiological data, our estimates reflect only the disease burden based on reported cases. Future research combining serological surveys with case surveillance is required to clarify age-related differences in infection risk versus symptomatic disease risk.

DENV-1 plays a dominant role in the dengue outbreaks in southern China. Villabona-Arenas C.J. et al. elucidated viral dispersal dynamics using Bayesian phylogeography with representative DENV-1 envelope gene sequences, revealing that DENV-1 strains collected in Guangdong Province, China during 1980 and 1998–2006 likely originated from Singapore, Thailand, and Vietnam (Villabona-Arenas and Zanotto, 2013). In mainland China, dengue epidemics are primarily attributed to imported cases. As major centers of cross-border trade in China, Guangdong and Yunnan provinces, particularly Guangzhou, Shantou, Zhanjiang, and Xishuangbanna, Ruili, and Jinghong, are vulnerable to importing cases from Southeast Asian nations, which spreads DENV to other cities in the provinces and across the nation. This assertion is supported by the fact that China’s border control measures during the Coronavirus Disease 2019(COVID-19) pandemic significantly reduced the dengue incidence in border provinces and cities (Jin et al., 2024). Between 2004 and 2013, over 80% of imported cases in China originated from South-East Asia, and these imported viruses continued to spread to the southern coastal provinces (Ning et al., 2015). Meanwhile, a study involving phylogenetic and Bayesian phylogeographic analyses of 200 DENV-1 genotype I dengue virus samples collected in the Asia-Pacific region between 2010 and 2014 and 2015 and 2019 found that Guangdong, Fujian, Zhejiang, Guangxi and Yunnan experienced frequent viral migration with Southeast Asian countries such as Thailand, Singapore, Vietnam and Indonesia between 2010 and 2019 (Yang et al., 2020). Furthermore, in terms of temporal trends, DENV-1 has remained the dominant serotype over the long term. Data from Zhao et al.’s study indicate that between 1990 and 2019, DENV-1 was reported in approximately 70% of the years, and occurred continuously from 2013 to 2019 (Zhao et al., 2024); although DENV-2 also occurred continuously during the same period, DENV-1 remained the predominant serotype. The four serotypes often co-circulate, for example in 2013 (serotypes 1, 2 and 3), 2016 (all four serotypes simultaneously) and 2019 (serotypes 1, 2 and 3), rather than simply ‘alternating dominance’ between serotypes.

The clinical symptoms observed in dengue patients are intricately connected to the disease’s underlying mechanisms. When the DENV infiltrates the human body, it triggers an immune response, resulting in the release of inflammatory cytokines. This cascade of events interferes with the body’s thermoregulatory center, ultimately causing fever. Simultaneously, the body’s attempts to dissipate heat leads to chills in patients. Additionally, non-structural protein 1 (NS1) engages macrophages through Toll-like receptor 4 (TLR4), contributing to endothelial cell damage and subsequent vascular leakage (Puerta-Guardo et al., 2016; Glasner et al., 2018; Biering et al., 2021; Li-hong et al., 2022). In patients with dengue who display hemorrhagic symptoms, there is a demethylation of the promoter genes for tumor necrosis factor alpha (TNF-α) and interferon gamma (IFN-γ). This process initiates an acute inflammatory response, leading to the development of a cytokine storm (Yang et al., 2024). These mechanisms explain why fever, asthenia, and headache are commonly observed in the clinical presentation of dengue patients. Laboratory findings indicate that DENV infection triggers intense immune responses, tissue damage, and hepatocyte injury in patients. There is a significant association between specific DENV serotypes and distinct clinical manifestations. This association has been widely documented in previous studies. For instance, an analysis of 362 laboratory-confirmed dengue cases in Rio de Janeiro revealed that patients infected with DENV-3 were more prone to shock and rash than those infected with DENV-2, and DENV-3 infection was associated with a higher incidence of abdominal pain and rash compared with DENV-1 (Passos et al., 2004). Another multicenter passive surveillance study conducted in four South American countries (Peru, Bolivia, Ecuador, and Paraguay) further demonstrated that DENV-3 infection was linked to a higher proportion of musculoskeletal and gastrointestinal symptoms, whereas DENV-4 infection more frequently presented with respiratory and cutaneous manifestations (Halsey et al., 2012). The complex interaction between the genetic characteristics of the virus and the host’s serotype-specific immune status jointly determines the severity of clinical manifestations in patients with dengue fever. From a viral perspective, differences in virulence attributable to genomic sequence variations exist among different serotypes and even among different genotypes within the same serotype. Samune et al. demonstrated through single-round infectious particle (SRIP) assays that the pre-membrane/envelope (prM/E) proteins of the DENV-2 Cosmopolitan genotype increased the infectivity of reporter virus particles to approximately three times that of the DENV-2 Asian-I genotype. Replicon assays confirmed that replacing the NS1-NS2B region of the DENV-2 Asian-I genotype with that of the Cosmopolitan genotype significantly enhanced viral RNA replication efficiency, whereas the reverse substitution reduced replication by approximately 26.8%. In an interferon receptor knockout (IFNR-KO) mouse model, chimeric viruses carrying both Cosmopolitan-type structural and NS1-NS2B proteins induced higher blood viral loads and mortality, confirming that sequence variation in these two regions jointly determines *in vivo* pathogenicity (Samune et al., 2024). Furthermore, studies by Thomas L and Yung CF found that the severity of clinical symptoms was significantly correlated with differences in DENV replication capacity (Thomas et al., 2008; Yung et al., 2015). These mechanistic insights explain why distinct genotypes within the same serotype can lead to markedly divergent clinical outcomes. For example, the Asian genotype of DENV-2, which is prevalent in Americas, the South Pacific and Sri Lanka, is significantly associated with severe dengue, whereas the Cosmopolitan genotype of DENV-2 which is prevalent in Singapore usually causes milder clinical manifestations (Gubler, 1998; Leitmeyer et al., 1999; Díaz et al., 2006; Yung et al., 2015). Meanwhile, the clinical presentation of dengue is not determined solely by viral characteristics; the host itself (e.g., genetics, immune status) also plays a key role. For example, a study of Dengue Hemorrhagic Fever (DHF) and Dengue fever patients in Venezuela found that patients with a low interleukin-10 (IL-10) genotype combined with a high TNF-α genotype all developed DHF (OR = 17.4 compared with Dengue fever, *p* = 0.017; OR = 19.5, *p* = 0.013, although this did not reach statistical significance after correction for multiple comparisons), suggesting that genetically determined low levels of anti-inflammatory cytokines and high levels of TNF-α may be involved in the pathogenic mechanism of DENV (Coffey et al., 2009). The theory of ADE posits that, at certain concentrations, specific antibodies bind to but do not neutralize viral particles from subsequent DENV infections. These virus-antibody complexes are recognized by Fcγ receptors, facilitating viral entry into target immune cells and enabling replication. This triggers an immune cascade leading to vascular leakage and severe dengue symptoms (Beatty et al., 2015; Guzman and Harris, 2015). Federico Narvaez’s research found that almost all severe cases occurred in patients with secondary infections (Narvaez et al., 2025). In summary, the clinical manifestations of dengue fever arise from complex interactions between viral characteristics and host factors. Differences in viral serotype/genotype can directly influence the severity and clinical phenotype of the disease by modulating viral replicative capacity and pathogenicity; conversely, host genetic polymorphisms and immune status regulate the intensity and direction of the immune response. The relative importance of these two factors may vary across different populations and geographical regions, offering some insight into the inconsistencies observed in the association between serotypes and clinical manifestations in global studies. Due to the notably low infection rate of DENV-4, there may be insufficient statistical power, which led to the identification of only one study addressing DENV-4 and its clinical manifestations. This limited data was inadequate for creating a forest plot, resulting in the exclusion of DENV-4 from the analysis of the relationship between DENV serotypes and clinical manifestations in this research.

Climate and population mobility are key factors influencing the distribution and characteristics of dengue outbreaks. In China, the temperate continental and monsoon climate, which features high temperatures and significant rainfall from August to October, provides ideal conditions for the proliferation of *Aedes aegypti* (*Ae. aegypti*), the primary vector responsible for DENV (Wang et al., 2022; Katzelnick et al., 2023; Jin et al., 2024). Rainfall offers *Aedes* a rich supply of organic matter. As noted by Lambrechts et al., elevated temperatures can enhance viral replication, which shortens the virus’s exogenous incubation period in the vector. This acceleration may lead to an increase in the incidence of dengue epidemics (Lambrechts et al., 2011). Secondly, the peak travel and business seasons in our country span from August to October. Consequently, the increased risk of imported dengue cases elevates the potential for local transmission. Dengue incidence in China is particularly high during this period due to these combined factors. **Figure 5** shows a shift in the onset of dengue cases in China from September to October. This delay may be attributable to climate change; rising temperatures significantly increase vector activity during the autumn months (September–October), extending the transmission window. Zhou et al. predicted that rising temperatures would prolong mosquito activity periods (Zhou et al., 2025). While an expected surge in *Ae. aegypti* populations may elevate dengue incidence, peaking between October and November, this projected timing differs from the temporal shift observed in this study. However, it does provide valuable insight into the mechanism behind this temporal lag.

The findings presented here regarding the northward spread of dengue cases offer a comprehensive analysis of the disease’s expansion patterns in China, consistent with various theoretical models. Firstly, China’s diverse geography spans tropical, subtropical, and temperate zones from south to north. Secondly, environmental factors play a crucial role; studies indicate that as diurnal temperature variations increase, the likelihood of dengue outbreaks in temperate areas also rises (Cheng et al., 2025). Research shows a significant correlation between the localized distribution of dengue outbreaks and the presence of DENV vectors in China. Human infections are primarily transmitted by mosquitoes of the *Aedes* genus. As global temperatures rise, subtropical and temperate regions are becoming more hospitable to *Aedes* populations, which in turn expands their geographical range and seasonal activity. A study investigating the global expansion and redistribution of *Aedes* mosquito transmission risks in the context of climate change revealed significant findings. Researchers discovered that the combined effects of global climate shifts and the adaptive evolution of mosquitoes have rendered certain tropical regions inhospitable for *Aedes* survival due to extreme temperatures. At the same time, *Aedes albopictus* (*Ae. albopictus*) has successfully established populations across a wider range of altitudinal gradients. In warmer regions, these populations no longer require hibernation, while those in temperate areas can endure cold winters by entering a state of dormancy. Furthermore, urban microenvironments offer warmer conditions than natural habitats, enhancing the survival prospects of *Ae. Aegypti* mosquitoes that have adapted to urban settings. The ongoing trend of urbanization in China is expected to exacerbate this situation (Liao et al., 2023). The northward spread of dengue cases within our country is fundamentally rooted in environmental factors. Mosquitoes, being ectothermic organisms, are significantly affected by ambient temperatures, which in turn influence their body temperature and various life processes such as survival, adult lifespan, larval development, and vector competence. Temperature plays a crucial role in the hatching of mosquito eggs. Research shows that *Ae. aegypti* larvae in temperate regions have adapted to cooler conditions. Studies reveal that these temperate *Ae. aegypti* hatch more quickly at temperatures of 5°C, 12°C, and 15°C, enhancing the potential for their spread to higher latitudes and colder climates (Nik Abdull Halim et al., 2022). Human activities significantly influence the occurrence of dengue in temperate regions. Due to the limited flight range of vector organisms, the movement of populations is crucial for the spread of DENV. Activities such as tourism can introduce the virus into areas with lower immunity levels, potentially leading to localized outbreak. For instance, in 2013, Xuchang City in Henan Province experienced its first outbreak of dengue. According to epidemiological investigations, individuals from the affected village had previously worked in Laos, a region where dengue is endemic (Huang et al., 2014). As socio-economic and cultural development progresses, the influence of population mobility on the transmission dynamics of DENV is expected to grow significantly. Researchers have employed representative concentration pathways to forecast future risk distribution maps for dengue in China. Their findings indicate that, compared to the high-risk dengue areas encompassing 142 counties and impacting 168 million residents during the 1981–2016 climate period, the high-risk zones for dengue in China are projected to expand throughout the 21st century under similar RCP scenarios. The only exception to this trend is RCP2.6, which indicates a turning point in the 2050s (Fan and Liu, 2019). During the transition from the late 20th century to the early 21st century, the primary habitats of *Ae. albopictus* and *Ae. aegypti* in China were predominantly found in the southern provinces, particularly Guangxi, Hainan, and Guangdong (Yue et al., 2022). In the study, it was found that under the influence of global warming, the suitable habitat distribution of *Ae. albopictus* in China is gradually shifting towards the northwest and northeast (Liu, 2023). In conclusion, climate change and associated temporal shifts are projected to expand the potential DENV transmission zones in China northward, shifting them from lower to higher latitudes.

Our study has several limitations. Firstly, the data utilized were sourced from published articles, which may have led to unrecorded cases of dengue. Additionally, since dengue is a self-limiting disease and some infections remain asymptomatic, it is likely that many cases went undetected. This suggests that the actual number of infections could be significantly higher than what is reflected in the recorded data. The search terms used did not specify individual Chinese cities, which led to the omission of articles using city names in the title. As a result, the study’s findings may not represent the entire territory of China. Secondly, the risk of bias assessment indicated that all included studies met the criteria for five key items: detailed descriptions of study subjects and settings, sufficient coverage in data analysis of the identified sample, the use of objective and standard criteria for measuring the condition, reliable measurement of the condition, and appropriate statistical analysis. However, all 11 articles (30.6%) failed to adequately address important confounding factors or perform subgroup analyses to control these variables, as outlined in the criterion regarding the identification and accounting of confounding factors and differences. This oversight implies that nearly one-third of the studies may have been influenced by confounding bias, thereby diminishing the reliability of their results. Thirdly, the conclusions of this study regarding the association between DENV serotypes and clinical manifestations are based solely on statistical descriptions of the included literature, rather than reflecting a direct causal relationship with the virus’s inherent pathogenicity. A variety of confounding factors may significantly influence the observed associations; for example, the circulating strains vary across different years of dengue outbreaks, and the level of herd immunity within the population is subject to dynamic changes. Furthermore, there was a lack of standardization in the criteria used by hospitals and other institutions to define clinical symptoms such as rash, diarrhea and headache when including cases in the study; the resulting bias may have led to a misinterpretation of the true association between specific serotypes and symptoms. Fourthly, in the studies we included, severe dengue accounted for only 1.83% of the pooled cases; this finding should be interpreted with caution. As the vast majority of reported cases were mild, field investigators rarely collected or reported detailed clinical and laboratory parameters needed to stratify severe cases according to confounding factors (age, comorbidities, and secondary infections). Consequently, this study cannot answer the question of ‘ which population groups in China face the highest risk of severe dengue’ —a gap that must be addressed through future prospective studies utilizing standardized case report forms. Fifthly, due to the absence of detailed population-level baseline data on occupational and age structures, the distributions of occupation and age reported in this study reflect only the composition of notified dengue cases, and should not be interpreted as indicating differential infection risk across these subgroups. Consequently, we could not determine whether specific occupational or age groups face elevated susceptibility to dengue. Further thematic investigations with appropriate population denominator data are warranted to validate any potential links between occupation, age, and dengue incidence. Lastly, we were unable to obtain complete data from the CPC for the period of 2005 to 2023. As a result, our analysis of the spatial distribution trends for dengue cases during this timeframe is not comprehensive. This is particularly true for the years 2020–2023, for which data are lacking, as mainland China was concurrently experiencing the novel coronavirus pandemic. This data gap prevents us from examining how the COVID-19 pandemic might have influenced the temporal distribution of dengue outbreaks or the northward spatial shift trend.

Nonetheless, we believe that our study effectively highlights the population-based epidemiological characteristics of dengue in China from 2005 to 2023. Given the occupational distribution and age range of dengue fever patients, prevention and control efforts should prioritize these groups. Furthermore, the widespread presence of DENV-1 in China emphasizes the importance of preventing the virus’s importation from neighboring countries, especially those in Southeast Asia, and highlights the need for collaborative vaccine development with these nations to jointly prevent dengue outbreaks. Additionally, the relationship between various DENV serotypes and patients’ clinical manifestations aids in serotype identification and allows for the evaluation of vaccine efficacy against different serotypes during both development and application. The temporal distribution of dengue cases in China reveals that cases primarily occur between August and October, which provides an opportunity to implement targeted epidemic control measures. Enhancing monitoring at transport hubs during this peak period is crucial to preventing the epidemic from worsening. Understanding the trend of dengue case’s northward expansion in China is critical for devising effective control strategies. High-risk areas should prioritize mosquito vector control and ensure sufficient reserves of medical supplies and personnel, while low-risk areas should concentrate on monitoring for potential epidemic spread.

## Data Availability

The original contributions presented in the study are included in the article/**Supplementary Material**. Further inquiries can be directed to the corresponding author.
