# Supplementary material for: Dengue outbreaks in China during 2005-2023: a systematic analysis
Source: Front Cell Infect Microbiol. 2026 Aug 18;16:1920586. doi: 10.3389/fcimb.2026.1920586 (PMC13529551; doi:10.3389/fcimb.2026.1920586)
Supplement: Supplementary file 1 [file Table1.docx]

**Table S1**

JBI Critical Appraisal Checklist for Prevalence Studies

| Study | *Was the sample representative of the target population?** | *Were study participants recruited in an appropriate way?* **^†^** | *Was the sample size adequate?***^‡^** | *Were the study subjects and setting described in detail?* | *Is the data analysis conducted with sufficient coverage of the identified sample?* | *Were objective, standard criteria used for measurement of the condition?* | *Was the condition measured reliably?***^§^** | *Was there appropriate statistical analysis?* | *Are all important confounding factors/ subgroups/ differences identified and accounted for?***^¶^** | *Were subpopulations identified using objective criteria?* | Total  Score |
| --- | --- | --- | --- | --- | --- | --- | --- | --- | --- | --- | --- |
| HUANG Ya-lan 2016[1] | Yes | Yes | Yes | Yes | Yes | Yes | Yes | Yes | No | Yes | 9 |
| CAI Xiao-li 2015[2] | Yes | Yes | Yes | Yes | Yes | Yes | Yes | Yes | u/c | Yes | 9 |
| FENG Yun 2014[3] | Yes | Yes | Yes | Yes | Yes | Yes | Yes | Yes | u/c | Yes | 9 |
| YANG Fang-ji 2015[4] | Yes | Yes | Yes | Yes | Yes | Yes | Yes | Yes | u/c | Yes | 9 |
| ZHENG Xiu-jun 2016[5] | Yes | Yes | Yes | Yes | Yes | Yes | Yes | Yes | u/c | Yes | 9 |
| CUI Xin-guo 2017[6] | Yes | Yes | Yes | Yes | Yes | Yes | Yes | Yes | u/c | Yes | 9 |
| WANG Xing 2018[7] | Yes | Yes | Yes | Yes | Yes | Yes | Yes | Yes | No | Yes | 9 |
| YANG Rong-xing 2019[8] | Yes | Yes | Yes | Yes | Yes | Yes | Yes | Yes | No | Yes | 9 |
| CAO Jia-qi 2019[9] | Yes | Yes | Yes | Yes | Yes | Yes | Yes | Yes | Yes | Yes | 10 |
| LI Man 2019[10] | Yes | Yes | Yes | Yes | Yes | Yes | Yes | Yes | Yes | Yes | 10 |
| FENG Shi-jun 2020[11] | Yes | Yes | Yes | Yes | Yes | Yes | Yes | Yes | Yes | Yes | 10 |
| LU Hong-yan 2020[12] | Yes | Yes | Yes | Yes | Yes | Yes | Yes | Yes | Yes | Yes | 10 |
| GUO Chuan 2020[13] | Yes | Yes | Yes | Yes | Yes | Yes | Yes | Yes | Yes | Yes | 10 |
| GAO Han-bing 2021[14] | Yes | Yes | Yes | Yes | Yes | Yes | Yes | Yes | Yes | Yes | 10 |
| LI Yang-si-qi 2021[15] | Yes | Yes | Yes | Yes | Yes | Yes | Yes | Yes | No | Yes | 9 |
| JIA Wen-shuang 2021[16] | Yes | Yes | Yes | Yes | Yes | Yes | Yes | Yes | No | Yes | 9 |
| LIN Yi-xiong 2021[17] | Yes | Yes | Yes | Yes | Yes | Yes | Yes | Yes | Yes | Yes | 10 |
| FANG Qi 2021[18] | Yes | Yes | Yes | Yes | Yes | Yes | Yes | Yes | Yes | Yes | 10 |
| WEN Yan-ping 2021[19] | Yes | Yes | Yes | Yes | Yes | Yes | Yes | Yes | Yes | Yes | 10 |
| WANG Xue-fei 2022[20] | Yes | Yes | Yes | Yes | Yes | Yes | Yes | Yes | Yes | Yes | 10 |
| JIANG Li-yun 2022[21] | Yes | Yes | Yes | Yes | Yes | Yes | Yes | Yes | Yes | Yes | 10 |
| CAI Lian-ru 2023[22] | Yes | Yes | Yes | Yes | Yes | Yes | Yes | Yes | Yes | Yes | 10 |
| YANG Fan 2023[23] | Yes | Yes | Yes | Yes | Yes | Yes | Yes | Yes | Yes | Yes | 10 |
| JIANG Li-yun 2024[24] | Yes | Yes | Yes | Yes | Yes | Yes | Yes | Yes | Yes | Yes | 10 |
| SONG Ji-jun 2024[25] | No | Yes | No | Yes | Yes | Yes | Yes | Yes | Yes | Yes | 8 |
| GUO Ting 2024[26] | No | Yes | Yes | Yes | Yes | Yes | Yes | Yes | Yes | Yes | 9 |
| GUO Xiaofang 2025[27] | Yes | Yes | Yes | Yes | Yes | Yes | Yes | Yes | Yes | Yes | 10 |
| XIE Zheng-hua 2019[28] | Yes | Yes | u/c | Yes | Yes | Yes | Yes | Yes | No | No | 7 |
| HuaYu 2019[29] | Yes | Yes | Yes | Yes | Yes | Yes | Yes | Yes | No | No | 8 |
| Qin-Long Jing 2012[30] | Yes | Yes | Yes | Yes | Yes | Yes | Yes | Yes | No | No | 8 |
| Jian-Peng Xiao 2016[31] | Yes | Yes | Yes | Yes | Yes | Yes | Yes | Yes | Yes | Yes | 10 |
| GUO Chuan 2020[32] | Yes | Yes | Yes | Yes | Yes | Yes | Yes | Yes | No | Yes | 9 |
| Yong Ping Lin 2016[33] | Yes | Yes | Yes | Yes | Yes | Yes | Yes | Yes | No | Yes | 9 |
| Liyun Jiang 2023[34] | Yes | Yes | Yes | Yes | Yes | Yes | Yes | Yes | Yes | Yes | 10 |
| Jiaqi Cao 2019[35] | Yes | Yes | Yes | Yes | Yes | Yes | Yes | Yes | Yes | Yes | 10 |
| Jin Huang 2024[36] | No | u/c | Yes | Yes | Yes | Yes | Yes | Yes | No | Yes | 7 |

* A study was considered to represent its target population if the basic sample characteristics of this study were found to mimic the basic characteristics of the targeted population and sub-populations.

**^†^** Random (probability) sampling was considered as the proper recruitment technique.

**^‡^** The sample size had to be calculated by the authors of each study, as the calculation of a universally-adequate sample size was considered to be unreliable due to the wide variability in the targeted population or sub-population of each study. If no sample size calculations were reported, “unclear” was given as an answer to this question.

**^§^** The following diagnostic techniques were decided to provide a reliable measurement of DENV sero-prevalence: Enzyme-Linked Immuno-Sorbent Assay (ELISA), ImmunoFluorescence Assay (IFA), Reverse-Transcriptase Polymerase Chain Reaction (RT-PCR) and Viral Neutralization Tests (VNT).

**^¶^** Important confounding factors were considered to be properly addressed if the study findings were confirmed by PCR or an investigation for cross-reactivity against other arboviral infection markers was conducted.

**Table S2**

Literature Information Table for Different Research Groups

| **Research Project** | **Included literature** | **The methods used to decide which results to collect** | **The process for determining which studies meet the criteria for each systematic review** | **Outcome indicator** |
| --- | --- | --- | --- | --- |
| Male-to-female ratio | 1，2，5-7，11，12，14-16，19，20，22，24-26，29-31，34，36 | Include data with clearly defined statistical figures and gender ratios. | Compile a table of research characteristics that meet the requirements and screen the data according to the descriptions in the article, excluding any data that does not meet the requirements or is duplicated. | Sex ratio |
| Occupational composition of dengue patients | 20，22，24，31，36 | Data incorporating clearly defined statistical counts where the grouping of the occupational is consistent across different literature sources. | Compile statistical data on the occupational distribution of dengue patients from various literature sources into a table, excluding data with inconsistent occupational classifications. | Percentage of each occupation within the sample |
| Age distribution of dengue patients | 5，12，20，24，31，36 | Data incorporating clearly defined statistical counts where the grouping of age variables is consistent across different literature sources. | Compile statistical data on the age distribution of dengue patients from various literature sources into a table, excluding data where age group classifications are inconsistent. | Percentage of each age group within the sample |
| Prevalence rates of each dengue serotype | 1，10，13-19，21-23，25，27 | Include data with clearly defined sample sizes and explicit serotype classification for dengue virus. | Data on the various dengue serotypes detected in different literature sources were collated into a table. Due to the limited availability of data on secondary infections, this study excluded serotype data pertaining to secondary dengue infections. | Percentage of each dengue serotype in the sample |
| Clinical characteristics and laboratory findings in patients with dengue fever | 5-9，11，28 | The included literature contained detailed samples and identical clinical symptoms. | Compile data on clinical manifestations observed in dengue patients across various literature sources into a table, excluding data with inconsistent indicators. | Percentage of clinical manifestations in dengue patients within the sample |
| Association between DENV serotypes and clinical manifestations in patients | 9，10，15-16 | The literature meticulously documents the number of dengue patients exhibiting distinct clinical manifestations across different serotypes, whilst studies incorporating dengue viruses of the same serotype encompass identical categories of clinical presentation. | Data from studies describing the association between dengue serotypes and clinical manifestations across various literature sources were collated into a table. As research on DENV-4 is limited, DENV-4 was excluded. | The “OR” of each dengue serotype to clinical manifestations |

Data from 4 was overwritten by 9; data from 32, 33, and 35 was overwritten by 6 and 34. Data from 3 serves as a reference source for imported cases. Therefore, although the content of each literature source differs, all were incorporated into the study. However, the five articles—3, 4, 32, 33, and 35—are not mentioned in the table above and are provided solely as supplementary data references.

**Table S3**

The table of Basic Information for Included Literature

| **Title** | **The Article's Basic Information** | **Participant characteristics** | **Intervention Details** | **Funding sources** |
| --- | --- | --- | --- | --- |
| HUANG Ya-lan 2016[1] | J Trop Med，Feb． 2016，Vol．16，No．2 | Source of cases: Suspected dengue fever cases reported by district-level disease control centres and hospitals across Shenzhen in 2014, totalling 452 cases. | Diagnostic criteria: Based on the Ministry of Health's "Diagnostic Criteria for Dengue Fever WS216-2008" | Undeclared |
| CAI Xiao-li 2015[2] | J Trop Med, Apr. 2015, Vol.15, No.4 | Source of cases: Patients presenting with fever at Guangzhou Eighth People's Hospital between August and October 2013, clinically diagnosed with dengue fever. | Diagnostic criteria:The industry standard of Chinese Ministry of Health．Diagnostic criteria and principles of management of dengue fever （WS 216- 2001） | Key Project of Guangzhou Municipal Health Commission（2009-Zhi-06）  Science & Technology Benefiting People and Smart City Special Project（2014Y2-00185） |
| FENG Yun 2014[3] | Chin J Viral Dis, October 2014, Vol.4 No.4 | Source of cases: Confirmed dengue fever cases in Ruili City from August to November 2013. | Diagnostic criteria: Based on the Ministry of Health's "Diagnostic Criteria for Dengue Fever WS216-2008" | China-US Collaborative Programme on Emerging and Re-emerging Infectious Diseases（U19-GH000004） |
| YANG Fang-ji 2015[4] | This article is a conference summary (informal journal paper), published in China CNKI. | Source of cases: Hospitalised patients diagnosed with dengue fever admitted to the Third Affiliated Hospital of Sun Yat-sen University during the dengue fever outbreak in Guangzhou from 1 October to 31 October 2014. | Retrospective study, no intervention measures | Undeclared |
| ZHENG Xiu-jun 2016[5] | Master's Thesis | Source of cases: Dengue fever cases admitted to the infectious diseases departments of major hospitals in Zhuhai from 2007 to 2013. | Retrospective study, no intervention measures | Zhuhai Medical Research Fund Project (No. 2015J036) |
| CUI Xin-guo 2017[6] | Master's Thesis | 2013–2015: 1,009 hospitalised cases of dengue fever. | Observe clinical symptoms and signs in accordance with WHO guidelines. | Yunnan Provincial Collaborative Innovation Center for Public Health and Disease Control(Project Code: 2014YNPHXT03)  Project Supported by National Natural Science Foundation of China（No.81160357, 30960327, 30660160)  Sino-British fellowship trust in UK, 313669) |
| WANG Xing 2018[7] | Master's Thesis | 1,005 cases admitted and diagnosed in the Infectious Diseases Department of Ruili People's Hospital from August 2013 to November 2016 | Retrospective study, no intervention measures | Undeclared |
| YANG Rong-xing 2019[8] | Chin J Hyg Insect ＆ Equip Apr 2019 Vol． 25 No． 2 | 47 hospitalised dengue fever patients admitted to hospitals in Nanshan District between September and October 2014. | Retrospective study, no intervention measures | Supported by the Guangdong Medical Research Fund (No. B2018113) |
| CAO Jia-qi 2019[9] | Master's Thesis | Guangzhou City: 28 acute-phase serum samples from suspected dengue fever patients collected in October 2014 from the Third Affiliated Hospital of Sun Yat-sen University.  Xishuangbanna Dai Autonomous Prefecture: 105 acute-phase serum samples from suspected dengue fever patients collected in November 2015 from Jinghong Farm Workers' Hospital and Xishuangbanna Mental Health Centre. | This study is observational in nature (involving molecular epidemiology and clinical characteristic analysis) and does not involve any experimental interventions. | Undeclared |
| LI Man 2019[10] | Master's Thesis | Hospitalised patients with dengue fever (DF) in Ruili City, Yunnan Province, 2014–2017. | Retrospective study, no intervention measures | Undeclared |
| FENG Shi-jun 2020[11] | Chin J Biologicals April 2020，Vol. 33 No. 4 | In September 2018, 96 hospitalised cases of dengue fever were admitted to various hospitals in Qiyang County, Yongzhou City, Hunan Province. | Retrospective study, no intervention measures | Yunnan Provincial Basic Research Program（No.2016FA029） |
| LU Hong-yan 2020[12] | Parasitoses and Infectious Diseases June 2020，Vol. 18，No.2 | From January to December 2019, 1,161 suspected dengue fever cases were submitted for testing by the disease control centres of various counties and districts in Nanning City. | Retrospective study, no intervention measures | Undeclared |
| GUO Chuan 2020[13] | Modern Preventive Medicine, 2020, Vol. 47, NO. 17 | 174 suspected dengue fever cases admitted to monitored hospitals in Shantou City between 2015 and 2017. | Retrospective study, no intervention measures | Shantou Municipal Science and Technology Plan Project（Shannan Ke [2018] No. 155） |
| GAO Han-bing 2021[14] | Chin J Health Lab Tec，Feb． 2021，Vol． 31，No． 4 | June 2017–June 2019: 38 confirmed cases of dengue fever at a hospital in Hangzhou, Zhejiang Province. | Retrospective study, no intervention measures | Undeclared |
| LI Yang-si-qi 2021[15] | Master's Thesis | Dengue fever patients in Gengma County, Lincang City, Yunnan Province, 2019 | Observational study (no intervention). | Project Supported by National Natural Science Foundation of China（No**.**U1602223）  Yunnan Provincial Major Science and Technology Special Program (No. 2017ZF007) |
| JIA Wen-shuang 2021[16] | Master's Thesis | 481 cases of dengue fever among inpatients at Jinghong Infectious Disease Hospital, Yunnan Province, 2019 | Observational study (no intervention). | Undeclared |
| LIN Yi-xiong 2021[17] | Journal of Pathogen Biology | Between 2015 and 2020, Zhuhai City recorded a total of 156 confirmed cases of dengue fever | This study is a molecular epidemiological investigation and does not involve clinical intervention measures. | Supported by Zhuhai Medical and Health Science and Technology Plan Project (Major Project) (No. ZH2201200004HJL)  Zhuhai Emergency Science and Technology Special Project for COVID-19 Prevention and Control (No. ZH22036302200052PWC) |
| FANG Qi 2021[18] | Journal of Tropical Medicine | Acute-phase serum samples from suspected and clinically diagnosed dengue fever cases in Jieyang City, 2014–2019, totalling 896 specimens | This study is laboratory-based research and does not involve clinical intervention. | Undeclared |
| WEN Yan-ping 2021[19] | Chinese Journal of Zoonoses | 212 cases (158 imported cases, 54 locally transmitted cases) | This study is a molecular epidemiological investigation and does not involve clinical intervention measures. | Supported by the Zhejiang Provincial Health Commission’s Medical Health Science and Technology Project (No.2020KY235, No.2019KY146) |
| WANG Xue-fei 2022[20] | Journal of Tropical Diseases and Parasitology | 400 imported dengue fever cases (55 laboratory-confirmed, 345 clinically diagnosed). | This paper presents an epidemiological and aetiological analysis and does not address clinical interventions. | Project Supported by National Natural Science Foundation of China(No.81560548) |
| JIANG Li-yun 2022[21] | Chinese Journal of Public Health | 1,655 cases (including 273 imported cases and 1,382 locally transmitted cases) | This paper presents an epidemiological and aetiological analysis and does not address clinical interventions. | Guangzhou Municipal Science and Technology Plan Project（No.20202030117）；  Guangzhou Municipal Key Discipline Project (No. 2021-2023-11)；  Guangzhou Municipal Health Science and Technology Project (No. 2021JA010049) |
| CAI Lian-ru 2023[22] | Anti Infect Pharm | 375 cases (no fatalities) | No clinical intervention was involved. | Jian Science and Technology Special Project (No. Jishi Kejianzi [2010] No. 7) |
| YANG Fan 2023[23] | Chinese Journal of Health Laboratory Technology | Cumulative total of 900 cases reported between 2015 and 2022 | No clinical intervention was involved. | Shenzhen Science and Technology Plan Project (No. JCYJ20190807103005636) ; Open Research Fund Program of the State Key Laboratory of Pathogen Biosecurity (No. SKLPBS2244) |
| JIANG Li-yun 2024[24] | Disease Surveillance | 2015–2019. 4,245 cases, data sourced from the Infectious Disease Reporting Information Management System of the Chinese Centre for Disease Control and Prevention | No clinical intervention was involved. | Supported by Guangzhou Science and Technology Plan Program, Key Laboratory Project, and Key Medicine Discipline Project |
| SONG Ji-jun 2024[25] | Modern Disease Control and Prevention | 22 confirmed cases of dengue fever (including two cases of concurrent malaria and dengue fever infection) | No clinical intervention was involved. | Joint Co-construction Project of Henan Medical Science and Technology Research Plan (No. LHGJ20191101) |
| GUO Ting 2024[26] | Infect Dis Info | Fuzhou reports 328 cases of dengue fever | No clinical intervention was involved. | Sub-project of National Key R&D Program of China (No. 2022YFC2305001) |
| GUO Xiaofang 2025[27] | China Tropical Medicine | 1,006 cases of dengue fever at national-level surveillance sites in Yunnan Province (Longchuan County, Menghai County, Hekou County) in 2023 | No clinical intervention was involved. | Project supported by National Natural Science Foundation of China (No. 81560548) |
| XIE Zheng-hua 2019[28] | Master's Thesis | 406 cases of dengue fever (from cases admitted to the Pearl River Hospital between 2015 and 2018) | No clinical intervention was involved. | National Science and Technology Major Project (No. 2017ZX10103011-006)。  Guangdong Provincial Science and Technology Plan Project (No. 2014A020212494) |
| HuaYu 2019[29] | Scientific Reports | July to November 2017, Hangzhou City, Zhejiang Province. Total number of cases: 1,138 locally transmitted cases. | Epidemiological investigation: conducted in accordance with the Guidelines for the Diagnosis and Treatment of Dengue Fever (2014, 2nd Edition) issued by the National Health Commission of China. | Hangzhou Municipal Active Design Research Project for Agriculture and Social Development (No. 2016013A03)  The Construction Fund of Key Medical Disciplines of Hangzhou (Public Health Laboratory, 2016-2018) |
| Qin-Long Jing 2012[30] | BMC Infectious Diseases | September to October 2010 Location: Jingtai Subdistrict Community, Yuexiu District, Guangzhou 1 imported case (Guangzhou resident returning from Thailand), 18 locally transmitted secondary cases | Diagnostic criteria: Based on the Ministry of Health's "Diagnostic Criteria for Dengue Fever WS216-2008" | Guangzhou Municipal Science and Technology Information Bureau (Grant No. 20091C161, Grant No 200402-D0041）  Science and Technology Plan Project of Guangzhou Health Bureau (Grant No. 201102A211003)  Science and Technology Plan Project of Department of Science and Technology of Guangdong Province (Grant No. 20108031600076)  National Natural Science Foundation of China (Grant No. 81172735, Grant No 309725） |
| Jian-Peng Xiao 2016[31] | Infectious Diseases of Poverty | In 2014, Guangdong Province reported a total of 45,224 cases of dengue fever, according to the National Centre for Disease Control and Prevention (NNIDRIS). | Case diagnosis is based on the unified criteria established by the Chinese Ministry of Health (clinical diagnosis or laboratory confirmation). | Guangdong Provincial Science and Technology Project Fundings(NO.2013A02029005、NO.2014A04040104、NO.2015A020213004) |
| Chuan GUO 2020[32] | Japanese Journal of Infectious Diseases | Serum samples from 174 patients collected by the Shantou Centre for Disease Control and Prevention from local hospitals | No clinical intervention was involved. | Medical Scientific Research Foundation of Guangdong Province(No.C2018007)  Science and Technology Planning Project of Shantou city |
| Yong Ping Lin 2016[33] | BMC Infectious Diseases | Inpatients with fever admitted during the 2014 dengue fever outbreak in Guangzhou.  Sample size: Suspected dengue fever cases: 387  Laboratory-confirmed dengue fever cases: 138 | No clinical intervention was involved. | Science and technology planned projects fund of Guangdong province;  BNP Paribas CBB fund |
| Liyun Jiang 2023[34] | Scientific Reports | Case data reported to the National Immunisation Disease Reporting System (NIDRS) between 2010 and 2019. | Case Definition and Diagnosis: In accordance with the Ministry of Health of China's Diagnostic Criteria for Dengue Fever (WS216-2008/2018) | Guangzhou Municipality Science and Technology Project(No.202002030117)  Guangzhou Municipal Health and Family Planning Commission(No.2021-2023-11, 2021BRP004) |
| Jiaqi Cao 2019[35] | PLOS ONE | 133 suspected dengue fever cases (28 in Guangzhou, 105 in Xishuangb anna) | No clinical intervention was involved. | CAMS Innovation Fund for Medical Sciences（No.2017-12M-3-017）;  National major instrument and equipment research and development program(No.2016YFF0103103);  Chinese Postdoctoral Science Foundation(No.2018MG30193);  Priority Project on Infectious Disease Control and Prevention(No.2018ZX10711001-004-001, 2018ZX10713002-001-008) |
| Jin Huang 2024[36] | BMC Infectious Diseases | This study was conducted by the Department of Infectious Diseases at the Second Affiliated Hospital of Fujian University of Traditional Chinese Medicine (also known as Fujian Provincial Second People's Hospital). The research data were sourced from the hospital's outpatient medical record system, incorporating clinical information from 251 patients. | Diagnostic criteria: Based on the 2009 WHO guidelines and China's Diagnostic Criteria for Dengue Fever (WS216-2018), encompassing both suspected and confirmed cases. | Undeclared |

**Table S4**

Literature that may have been included but was ultimately excluded

| **Title** | **Reason for exclusion** |
| --- | --- |
| XIONG Yi-Quan 2014[37] | Occupational distribution data is incomplete. |
| NING Dan 2017[38] | The age groupings in the article do not meet the inclusion criteria. |
| HUANG Ying 2017[39] | The sample size of the case data in the article is small. |
| FAN Jian-Hua 2017[40] | The age groupings in the article do not meet the inclusion criteria. |
| CHEN Zhong-Wei 2018[41] | The age groupings in the article do not meet the inclusion criteria. |
| FAN Jian-Hua 2019[42] | The age distribution and occupational distribution data for patients are not detailed. |
| CAI Liang 2019[43] | The age distribution is not detailed. |
| YE Ling 2020[44] | The sample size of the case data in the article is small. |
| LIN Yi-Xiong 2021[45] | The age groupings in the article do not meet the inclusion criteria. |
| TIAN Jie 2021[46] | The occupational distribution data for patients is incomplete and lacks detail. |
| YU Tingting 2022[47] | The age groupings in the article do not meet the inclusion criteria. |
| CUI Feng-Fu 2022[48] | The source of the cases in the text falls outside the timeframe of this study's design. |
| XIANG Wei-Ying 2024[49] | The sample size of the case data in the article is small. |
| L Huang 2016[50] | The outbreak reported in this article duplicates that already included in the article. |
| Ting-Song Hu 2017[51] | The article does not contain the data required by this research institute. |
| Jimin Sun 2017[52] | The article does not contain the data required by this research institute. |
| Shengjie Lai 2015[53] | The age groupings in the article do not meet the inclusion criteria. |
| Zhoubin Zhang 2019[54] | The age groupings in the article do not meet the inclusion criteria. |
| Jiangping Ren 2022[55] | Occupational classification data does not meet the requirements of this study. |
| Xinchang Lun 2022[56] | The article does not contain the data required by this research institute. |
| Q L Jing 2018[57] | The article does not contain the data required by this research institute. |

Table S5

The basic data for each research group

A:Male-to-female ratio

| **Study** | **Sample size** | **Sex ratio(male to female)** |
| --- | --- | --- |
| HUANG Ya-lan 2016[1] | 332 | 199：133 |
| CAI Xiao-li 2015[2] | 204 | 125：79 |
| ZHENG Xiu-jun 2016[5] | 164 | 83:81 |
| CUI Xin-guo 2017[6] | 1009 | 482:527 |
| WANG Xing 2018[7] | 1005 | 687:318 |
| FENG Shi-jun 2020[11] | 96 | 47：49 |
| LU Hong-yan 2020[12] | 915 | 440：475 |
| GAO Han-bing 2021[14] | 38 | 22:16 |
| LI Yang-si-qi 2021[15] | 140 | 70:70 |
| JIA Wen-shuang 2021[16] | 481 | 253:228 |
| WEN Yan-ping 2021[19] | 212 | 114:98 |
| WANG Xue-fei 2022[20] | 400 | 267:133 |
| CAI Lian-ru 2023[22] | 375 | 187:188 |
| JIANG Li-yun 2024[24] | 4245 | 2298:1947 |
| SONG Ji-jun 2024[25] | 22 | 18:4 |
| GUO Ting 2024[26] | 328 | 168:60 |
| HuaYu 2019[29] | 1138 | 570:568 |
| Qin-Long Jing 2012[30] | 18 | 8:10 |
| Jian-Peng Xiao 2016[31] | 45224 | 25035:20189 |
| Liyun Jiang 2023[34] | 44385 | 22126:22259 |
| Jin Huang 2024[36] | 251 | 124:127 |

B:Social attributes of dengue patients

(1)Occupational composition of dengue patients

| **Study** | **Sample size** | **House worker /unemployed** | **Worker** | **Student** | **Farmer** | **Business service** | **Administrative staff** | **Others** |
| --- | --- | --- | --- | --- | --- | --- | --- | --- |
| WANG Xue-fei 2022[20] | 400 | 78 | 43 | 22 | 46 | 56 | 52 | 103 |
| CAI Lian-ru 2023[22] | 375 | 7 | 67 | 37 | 117 | 96 | 14 | 37 |
| JIANG Li-yun 2024[24] | 4245 | 834 | 462 | 329 | 245 | 751 | 206 | 1418 |
| Jian-Peng Xiao 2016[31] | 45224 | 10472 | 4923 | 3360 | 2189 | 5723 | 1699 | 10968 |
| Jin Huang 2024[36] | 251 | 17 | 24 | 32 | 13 | 35 | 36 | 94 |

(2)Age distribution of dengue patients

| **Study** | **Sample size** | **0-9** | **10-19** | **20-29** | **30-39** | **40-49** | **50-59** | **≥60** |
| --- | --- | --- | --- | --- | --- | --- | --- | --- |
| ZHENG Xiu-jun 2016[5] | 164 | 11 | 19 | 44 | 36 | 23 | 22 | 9 |
| LU Hong-yan 2020[12] | 915 | 17 | 68 | 190 | 227 | 143 | 127 | 143 |
| WANG Xue-fei 2022[20] | 400 | 8 | 16 | 135 | 124 | 71 | 37 | 9 |
| JIANG Li-yun 2024[24] | 4245 | 109 | 253 | 997 | 922 | 738 | 568 | 658 |
| Jian-Peng Xiao 2016[31] | 44385 | 1899 | 3170 | 9608 | 8339 | 7745 | 5974 | 7650 |
| Jin Huang 2024[36] | 251 | 16 | 18 | 28 | 52 | 37 | 53 | 47 |

C:Prevalence rates of each dengue serotype

| **Study** | **Province** | **Sample size** | **DENV-1** | **DENV-2** | **DENV-3** | **DENV-4** |
| --- | --- | --- | --- | --- | --- | --- |
| HUANG Ya-lan 2016[1] | Guangdong | 267 | 236 | 24 | 1 | 6 |
| LIN Yi-xiong 2021[17] | Guangdong | 156 | 120 | 26 | 10 | 0 |
| FANG Qi 2021[18] | Guangdong | 556 | 534 | 18 | 3 | 1 |
| YANG Fan 2023[23] | Guangdong | 809 | 633 | 111 | 57 | 8 |
| JIANG Li-yun 2024[24] | Guangdong | 2342 | 1919 | 400 | 16 | 7 |
| GUO chuan 2020[32] | Guangdong | 27 | 10 | 15 | 1 | 1 |
| LI Man 2019[10] | Yunnan | 429 | 305 | 77 | 28 | 19 |
| LI Yang-si-qi 2021[15] | Yunnan | 141 | 33 | 43 | 64 | 1 |
| JIA Wen-shuang 2021[16] | Yunnan | 481 | 279 | 107 | 95 | 0 |
| GUO Xiaofang 2025[27] | Yunnan | 350 | 305 | 43 | 1 | 1 |
| GAO Han-bing 2021[14] | Zhejiang | 38 | 1 | 32 | 3 | 2 |
| WEN Yan-ping 2021[19] | Zhejiang | 178 | 127 | 34 | 12 | 5 |
| CAI Lian-ru 2023[22] | JIangxi | 375 | 215 | 92 | 34 | 34 |
| SONG Ji-jun 2024[25] | Henan | 17 | 11 | 5 | 1 | 0 |
| FENG Shi-jun 2020[11] | Hunan | 96 | 0 | 96 | 0 | 0 |

D:Clinical characteristics in patients with dengue fever

| **Study** | **Sample size** | **Cough** | **Nausea** | **Diarrhea** | **Anorexia** | **Vomiting** | **Asthenia** | **Fever** | **Headache** | **Rash** | **Myalgia** |
| --- | --- | --- | --- | --- | --- | --- | --- | --- | --- | --- | --- |
| ZHENG Xiu-jun 2016[5] | 164 | 18 | 52 | 21 | 118 | 28 | 121 | 164 | 113 | 118 | 96 |
| WANG Xing 2018[7] | 1005 | 33 | 181 | 172 | 653 | 181 | 854 | 1005 | 844 | 467 | 761 |
| XIE Zheng-hua 2019[28] | 406 | 34 | 46 | 36 | 44 | 32 | 138 | 392 | 101 | 126 | 110 |

laboratory findings in patients with dengue fever

| **Study** | **Sample size** | **AST↑(%)** | **ALT↑(%)** | **Sample size** | **WBC↓(%)** | **PLT↓(%)** |
| --- | --- | --- | --- | --- | --- | --- |
| ZHENG Xiu-jun 2016[5] | 163 | 74.52 | 45.22 | 163 | 93.25 | 90.18 |
| CUI Xin-guo 2017[6] | 342 | .80.1 | 69 | 346 | 79.2 | 86.7 |
| CUI Xin-guo 2017[6] | 662 | 82.3 | 66 | 663 | 86.6 | 86.4 |
| YANG Rong-xing 2019[8] | 47 | 25.5 | 21.2 | 47 | 97.8 | 83 |
| CAO Jia-qi 2019[9] | 57 | 91.2 | 89.5 | 57 | 68.4 | 66.7 |
| FENG Shi-jun 2020[11] | 96 | 45.8 | 16.7 | 96 | 78.1 | 55.2 |
| XIE Zheng-hua 2019[28] | 270 | 77 | 60.4 | 394 | 71.1 | 52.3 |

This article was cited twice due to inconsistent sample sizes when detecting different "laboratory indicators".

Distribution of dengue clinical severity across the included studies

| **Study** | **Sample size** | **Non-severe dengue cases** | **Severe dengue cases** | **Deaths** |
| --- | --- | --- | --- | --- |
| WANG Xing 2018[7] | 1005 | 1003 | 2 | 0 |
| FENG Shi-jun 2020[11] | 96 | 83 | 13 | 0 |
| LI Yang-si-qi 2021[15] | 141 | 140 | 1 | 0 |
| JIA Wen-shuang 2021[16] | 481 | 477 | 4 | 0 |
| SONG Ji-jun 2024[25] | 22 | 21 | 1 | 0 |
| GUO Ting 2024[26] | 328 | 317 | 11 | 0 |
| HuaYu 2019[29] | 1138 | 1138 | 0 | 0 |
| Liyun Jiang 2023[34] | 44385 | 43998 | 397 | 10 |

seropositivity

| **Study** | **Sample size** | **Ig-M(%)** | **Ig-G(%)** |
| --- | --- | --- | --- |
| HUANG Ya-lan 2016[1] | 332 | 48.80 | 16.27 |
| CAI Xiao-li 2015[2] | 204 | 94.61 | 23.04 |
| CAO Jia-qi 2019[9] | 112 | 82.14 | 76.79 |
| GUO Ting 2024[26] | 328 | 20.43 | 9.15 |
| XIE Zheng-hua 2019[28] | 1498 | 14.95 | 7.41 |

E:The date of “Association of DENV serotypes with clinical characterisation of patients”

| Study | Sample size | Characteristics |
| --- | --- | --- |
| CAO Jia-qi 2019[9] | 43 | **Study** \| **All(43)** \| **DENV-1(13)** \| **DENV-2(19)** \| **DENV-1&2(11)**; Headache \| 28（65.12%） \| 4（30.77%） \| 15（78.95%） \| 9（81.82%）; Abdominal pain \| 2（4.65%） \| 1（7.69%） \| 1（9.09%） \| 0; Anorexia \| 25（58.14%） \| 1（7.69%） \| 15（78.95%） \| 9（81.82%）; Nausea \| 25（58.14%） \| 1（7.69%） \| 15（78.95%） \| 9（81.82%）; Rash \| 7（16.28%） \| 2（15.38%） \| 4（21.05%） \| 1（9.09%）; Bleeding \| 7（16.28%） \| 6（46.15%） \| 0 \| 1（9.09%）; Vomiting \| 11（25.58%） \| 0 \| 7（36.84%） \| 4（36.36%）; Asthenia \| 23（53.49%） \| 3（23.08） \| 13（68.42%） \| 7（63.64%）; Diarrhea \| 5（11.63%） \| 2（15.38%） \| 2（10.53%） \| 1（9.09%） |
| LI Man 2019[10] | 15 | **Study** \| **All(150)** \| **DENV-1(75)** \| **DENV-2(57)** \| **DENV-3(9)** \| **DENV-4(9)**; Headache \| 113（75.33%） \| 56（74.67%） \| 45（78.95%） \| 6（66.67%） \| 6（66.67%）; Eye pain \| 10(6.67%) \| 6(2.67%) \| 4(7.02%) \| 0 \| 0; Anorexia \| 40(26.67%) \| 22(29.33%) \| 12(22.22%) \| 3(33.33%) \| 3(33.33%); Nausea \| 21(14%) \| 7(9.33%) \| 9(8.93%) \| 2(22.22%) \| 3(33.33%); Rash \| 56(37.33%) \| 37(49.33%) \| 11(19.3%) \| 8(88.89%) \| 0; Bleeding \| 13(8.67%) \| 11(14.67%) \| 2(3.5%) \| 0 \| 0; Vomiting \| 15(10%) \| 8(10.67%) \| 5(8.77%) \| 0 \| 2(22.22%); Asthenia \| 49(32.67%) \| 16(21.33%) \| 21(36.84%) \| 7(77.78%) \| 5(55.56%) |
| LI Yang-si-qi 2021[15] | 140 | **Study** \| **All(140)** \| **DENV-1(33)** \| **DENV-2(43)** \| **DENV-3(64)**; Headache \| 93(66.43%) \| 24(72.73) \| 30(69.77%) \| 39(60.94%); Anorexia \| 95(67.86%) \| 24(72.73) \| 27(62.79%) \| 44(68.75%); Nausea \| 16(11.43%) \| 4(12.12%) \| 4(9.3%) \| 8(12.5%); Abdominal pain \| 4(2.86%) \| 0 \| 2(4.65%) \| 2(3.13%); Vomiting \| 5(3.57%) \| 1(3.03%) \| 0 \| 4(6.25%); Asthenia \| 122(87.14%) \| 29(87.88%) \| 37(86.05%) \| 56(87.5%); Diarrhea \| 16(11.43%) \| 4(12.12%) \| 2(4.65%) \| 10(15.63%); Eye pain \| 10(7.14%) \| 1(3.03%) \| 2(4.65%) \| 7(10.94%) |
| JIA Wen-shuang 2021[16] | 481 | **Study** \| **All(481)** \| **DENV-1(279)** \| **DENV-2(107)** \| **DENV-3(95)**; Headache \| 423(87.9%) \| 253(90.7%) \| 93(86.9%) \| 77(81.1%); Anorexia \| 304(63.2%) \| 180(64.5%) \| 60(56.1%) \| 64(67.4%); Bleeding \| 87(18.1%) \| 57(20.4%) \| 20(18.7%) \| 10(10.5%); Nausea \| 197(41%) \| 120(43%) \| 47(43.9%) \| 30(31.6%); Abdominal pain \| 48(10%) \| 30(10.8%) \| 13(12.1%) \| 4(4.2%); Rash \| 253(52.6%) \| 158(56.6%) \| 51(47.7%) \| 44(46.3%); Vomiting \| 54(11.2%) \| 36(12.9%) \| 7(6.5%) \| 11(11.6%); Asthenia \| 405(84.2%) \| 245(87.8%) \| 81(75.7%) \| 79(83.2%); Diarrhea \| 154(32%) \| 108(38.7%) \| 29(27.1%) \| 17(17.9%); Eye pain \| 26(5.4%) \| 11(3.9%) \| 8(7.5%) \| 7(7.4%) |

Table S6

PRISMA_2020_checklist

| **Section and Topic** | **Item #** | **Checklist item** | **Location where item is reported** |
| --- | --- | --- | --- |
| **TITLE** | | |  |
| Title | 1 | Identify the report as a systematic review. | Title |
| **ABSTRACT** | | |  |
| Abstract | 2 | See the PRISMA 2020 for Abstracts checklist. | supplementary information |
| **INTRODUCTION** | | |  |
| Rationale | 3 | Describe the rationale for the review in the context of existing knowledge. | Background section in the abstract |
| Objectives | 4 | Provide an explicit statement of the objective(s) or question(s) the review addresses. | Conclusion section in the abstract |
| **METHODS** | | |  |
| Eligibility criteria | 5 | Specify the inclusion and exclusion criteria for the review and how studies were grouped for the syntheses. | Searching Strategy and Selection Criteria |
| Information sources | 6 | Specify all databases, registers, websites, organisations, reference lists and other sources searched or consulted to identify studies. Specify the date when each source was last searched or consulted. | Searching Strategy and Selection Criteria |
| Search strategy | 7 | Present the full search strategies for all databases, registers and websites, including any filters and limits used. | Searching Strategy and Selection Criteria |
| Selection process | 8 | Specify the methods used to decide whether a study met the inclusion criteria of the review, including how many reviewers screened each record and each report retrieved, whether they worked independently, and if applicable, details of automation tools used in the process. | Searching Strategy and Selection Criteria |
| Data collection process | 9 | Specify the methods used to collect data from reports, including how many reviewers collected data from each report, whether they worked independently, any processes for obtaining or confirming data from study investigators, and if applicable, details of automation tools used in the process. | Data Extraction and Analysis |
| Data items | 10a | List and define all outcomes for which data were sought. Specify whether all results that were compatible with each outcome domain in each study were sought (e.g. for all measures, time points, analyses), and if not, the methods used to decide which results to collect. | Table S2 |
|  | 10b | List and define all other variables for which data were sought (e.g. participant and intervention characteristics, funding sources). Describe any assumptions made about any missing or unclear information. | Table S2 |
| Study risk of bias assessment | 11 | Specify the methods used to assess risk of bias in the included studies, including details of the tool(s) used, how many reviewers assessed each study and whether they worked independently, and if applicable, details of automation tools used in the process. | Searching Strategy and Selection Criteria |
| Effect measures | 12 | Specify for each outcome the effect measure(s) (e.g. risk ratio, mean difference) used in the synthesis or presentation of results. | Table S2 |
| Synthesis methods | 13a | Describe the processes used to decide which studies were eligible for each synthesis (e.g. tabulating the study intervention characteristics and comparing against the planned groups for each synthesis (item #5)). | Table S2 |
|  | 13b | Describe any methods required to prepare the data for presentation or synthesis, such as handling of missing summary statistics, or data conversions. | In the parts of “Data Extraction and Analysis” and “Statistical methods” |
|  | 13c | Describe any methods used to tabulate or visually display results of individual studies and syntheses. | Data Extraction and Analysis |
|  | 13d | Describe any methods used to synthesize results and provide a rationale for the choice(s). If meta-analysis was performed, describe the model(s), method(s) to identify the presence and extent of statistical heterogeneity, and software package(s) used. | Statistical methods |
|  | 13e | Describe any methods used to explore possible causes of heterogeneity among study results (e.g. subgroup analysis, meta-regression). | NO |
|  | 13f | Describe any sensitivity analyses conducted to assess robustness of the synthesized results. | NO |
| Reporting bias assessment | 14 | Describe any methods used to assess risk of bias due to missing results in a synthesis (arising from reporting biases). | Searching Strategy and Selection Criteria |
| Certainty assessment | 15 | Describe any methods used to assess certainty (or confidence) in the body of evidence for an outcome. | Data Extraction and Analysis |
| **RESULTS** | | |  |
| Study selection | 16a | Describe the results of the search and selection process, from the number of records identified in the search to the number of studies included in the review, ideally using a flow diagram. | Fig1 in the results |
|  | 16b | Cite studies that might appear to meet the inclusion criteria, but which were excluded, and explain why they were excluded. | Table S4 |
| Study characteristics | 17 | Cite each included study and present its characteristics. | Table S3 |
| Risk of bias in studies | 18 | Present assessments of risk of bias for each included study. | Table S1 |
| Results of individual studies | 19 | For all outcomes, present, for each study: (a) summary statistics for each group (where appropriate) and (b) an effect estimate and its precision (e.g. confidence/credible interval), ideally using structured tables or plots. | Results |
| Results of syntheses | 20a | For each synthesis, briefly summarise the characteristics and risk of bias among contributing studies. | Table S5 |
|  | 20b | Present results of all statistical syntheses conducted. If meta-analysis was done, present for each the summary estimate and its precision (e.g. confidence/credible interval) and measures of statistical heterogeneity. If comparing groups, describe the direction of the effect. | Results |
|  | 20c | Present results of all investigations of possible causes of heterogeneity among study results. | Results |
|  | 20d | Present results of all sensitivity analyses conducted to assess the robustness of the synthesized results. | NO |
| Reporting biases | 21 | Present assessments of risk of bias due to missing results (arising from reporting biases) for each synthesis assessed. | NO |
| Certainty of evidence | 22 | Present assessments of certainty (or confidence) in the body of evidence for each outcome assessed. | Results |
| **DISCUSSION** | | |  |
| Discussion | 23a | Provide a general interpretation of the results in the context of other evidence. | Discussion |
|  | 23b | Discuss any limitations of the evidence included in the review. | Discussion |
|  | 23c | Discuss any limitations of the review processes used. | Discussion |
|  | 23d | Discuss implications of the results for practice, policy, and future research. | Discussion |
| **OTHER INFORMATION** | | |  |
| Registration and protocol | 24a | Provide registration information for the review, including register name and registration number, or state that the review was not registered. | The review was not registered |
|  | 24b | Indicate where the review protocol can be accessed, or state that a protocol was not prepared. | Protocol was not prepared. |
|  | 24c | Describe and explain any amendments to information provided at registration or in the protocol. | No |
| Support | 25 | Describe sources of financial or non-financial support for the review, and the role of the funders or sponsors in the review. | Funding |
| Competing interests | 26 | Declare any competing interests of review authors. | There is no competing interest among the review authors. |
| Availability of data, code and other materials | 27 | Report which of the following are publicly available and where they can be found: template data collection forms; data extracted from included studies; data used for all analyses; analytic code; any other materials used in the review. | The analysed data are presented in Table S5; the code will not be made publicly available. |

*From:* Page MJ, McKenzie JE, Bossuyt PM, Boutron I, Hoffmann TC, Mulrow CD, et al. The PRISMA 2020 statement: an updated guideline for reporting systematic reviews. BMJ 2021;372:n71. doi: 10.1136/bmj.n71. This work is licensed under CC BY 4.0. To view a copy of this license, visit <https://creativecommons.org/licenses/by/4.0/>

Table S7

PRISMA_2020_abstract_checklist

| **Section and Topic** | **Item #** | **Checklist item** | **Reported (Yes/No)** |
| --- | --- | --- | --- |
| **TITLE** | | |  |
| Title | 1 | Identify the report as a systematic review. | Yes |
| **BACKGROUND** | | |  |
| Objectives | 2 | Provide an explicit statement of the main objective(s) or question(s) the review addresses. | Yes |
| **METHODS** | | |  |
| Eligibility criteria | 3 | Specify the inclusion and exclusion criteria for the review. | Yes |
| Information sources | 4 | Specify the information sources (e.g. databases, registers) used to identify studies and the date when each was last searched. | Yes |
| Risk of bias | 5 | Specify the methods used to assess risk of bias in the included studies. | Yes |
| Synthesis of results | 6 | Specify the methods used to present and synthesise results. | Yes |
| **RESULTS** | | |  |
| Included studies | 7 | Give the total number of included studies and participants and summarise relevant characteristics of studies. | Yes |
| Synthesis of results | 8 | Present results for main outcomes, preferably indicating the number of included studies and participants for each. If meta-analysis was done, report the summary estimate and confidence/credible interval. If comparing groups, indicate the direction of the effect (i.e. which group is favoured). | Yes |
| **DISCUSSION** | | |  |
| Limitations of evidence | 9 | Provide a brief summary of the limitations of the evidence included in the review (e.g. study risk of bias, inconsistency and imprecision). | Yes |
| Interpretation | 10 | Provide a general interpretation of the results and important implications. | Yes |
| **OTHER** | | |  |
| Funding | 11 | Specify the primary source of funding for the review. | Yes |
| Registration | 12 | Provide the register name and registration number. | The review was not registered |

*From:* Page MJ, McKenzie JE, Bossuyt PM, Boutron I, Hoffmann TC, Mulrow CD, et al. The PRISMA 2020 statement: an updated guideline for reporting systematic reviews. BMJ 2021;372:n71. doi: 10.1136/bmj.n71. This work is licensed under CC BY 4.0. To view a copy of this license, visit <https://creativecommons.org/licenses/by/4.0/>

**References**

1.	Ya-lan H, Fan Y, Da-na H, Miao W, Yue L, Chun-li W, et al. Analysis on the etiological and epidemic characteristics of dengue fever in Shenzhen city in 2014. J Trop Med. 2016;16(02):131-5.

2.	Xiao-li C, Ling-zhai Z, Hua-li L, Shuang Q, Xiao-zhen Y, Wen-xin H, et al. Etiological characteristics of 204 patients with dengue fever. J Trop Med. 2015;15(04):484-6.

3.	Yun F, Yong-hua L, Zheng-liu Y, Ping L, Zhao-lan Y, Yu-zhen Z, et al. Molecular epidemiologic analysis of an outbreak of dengue fever in 2013 at Ruili city, Yunnan province of China. Chin J Viral Dis. 2014;4(04):306-11. doi: 10.16505/j.2095-0136.2014.04.006.

4.	Fang-ji Y, Ying-jie W, Yu-tian C, editors. [Clinical and Laboratory Characteristics Analysis of 47 Cases of DENV-1 Ordinary Dengue Fever Patients]. Proceedings of the Seventh National Conference on AIDS and Hepatitis C of the Chinese Medical Association; 2015; Shenzhen, China. Chinese.

5.	Xiu-jun Z. A Research on the Clinical Features and Genotype of Dengue Patients in Zhuhai City during 2007-2013 [Master’s Thesis]: Zunyi Medical University; 2016.

6.	Xin-guo C. Study on the clinical characteristics of dengue fever in Yunnan, Xishuangbanna prefecture, China [Master’s Thesis]: Dali University; 2017.

7.	Xing W. Study on the clinical feature of 1005 cases of Dengue fever in Ruili from 2013 to 2016 [Master’s Thesis]: Dali University; 2018.

8.	Rong-xing Y, Shao-hua Z, Liang-qiang L, Chuan-wen D. Analysis of clinical and laboratory features of 47 patients with dengue fever in Nanshan District of Shenzhen. Chin J Hyg Insect ＆ Equip. 2019;25(02):144-7. doi: 10.19821/j.1671-2781.2019.02.016.

9.	Jia-qi C. [Epidemiology and Clinical Features of Dengue Fever Molecular Epidemic in Guangzhou City and Xishuangbanna Prefecture from 2014 to 2015] [Master’s Thesis]: Peking Union Medical College; 2019. Chinese.

10.	Man L. Study on the genotype and Clinical features of Dengue Virus in Ruili City of Yunnan from 2014-20117 [Master's Thesis]: Dali University; 2019.

11.	Shi-jun F, Jiao-qiong G, Jun C, Qing R, Qiang-ming S. Clinical and laboratory characteristics of 96 cases of dengue fever in

Qiyang County，Hunan Province，China in 2018. Chin J Biologicals 2020;33(04):423-8+33. doi: 10.13200/j.cnki.cjb.003036.

12.	Hong-yan L, Hao N, Anda T, Cheng Y, Liu-jiang Y, Bing H. Surveillance and Analysis of Suspected Cases of Dengue Fever in Nanning City in 2019. Parasitoses and Infectious Diseases. 2020;18(02):75-8+83.

13.	Chuan G, Xiu-zhu Z, Xu-bin Z, Jie-min L, Zhi-hua Z, Wan C. Molecular epidemiology of Dengue virus, Shantou City, 2015-1017. Modern Preventive Medicine. 2020;47(17):3228-33. doi: 10.20043/j.cnki.mpm.2020.17.039.

14.	Han-bing G, Yong-le Z, Wei W. Analysis of laboratory test results for 38 Dengue patients in a hospital in Zhejiang Province. Chin J Health Lab Tec. 2021;31(04):414-6.

15.	Yang-si-qi L. Analysis of clinical characteristics, serotypes and genotypes of dengue fever in Gengma County of Yunnan province [Master’s Thesis]: Kunming Medical University; 2021.

16.	Wen-shuang J. Clinical characteristics of Dengue Fever in Jinghong, Yunnan province in 2019 [Master's Thesis]: Dali University; 2021.

17.	Yi-xiong L, Hui-tao H, Quan-de W, Wen-yan H. Analysis of the prevalence and genetic characteristics of dengue fever virus in Zhuhai from 2015 to 2020. Journal of Pathogen Biology. 2021;16(06):701-4. doi: 10.13350/j.cjpb.210617.

18.	Qi F, Geng-hui L, Jing Y, Gui-zhen F, Huan Z. Sequence analysis of E gene of dengue virus isolated in Jieyang，2014-2019. J Trop Med. 2021;21(07):844-8.

19.	Yan-ping W, Hao-qiu W, Xin-fen Y, Guo-zhong Z, Yin-yan Z, Xin Q. Molecular epidemiological analysis of Dengue virus in Hangzhou, 2019. Chinese Journal of Zoonoses. 2021;37(12):1096-101.

20.	Xue-fei W, Rong T, Zhi-hui C, Ming-dong Y, Xiao-fang G. Analysis on the epidemiological and etiological characteristics of imported dengue fever cases in Kunming in 2019. J Trop Dis Parasitol. 2022;20(04):215-20.

21.	Li-yun J, Yuan L, Wen-zhe S, Lei L, Yi-min C, Wen-hui L. Epidemiological features of dengue fever and envelope gene of dengue virus

among  imported  and  indigenous  cases  in  Guangzhou  city:  a  comparison

analysis on registry data of 2019. Chin J Public Health. 2022;38(11):1373-8.

22.	Lian-ru C, Xiang-mei N, Qiu-ying D. Epidemiological Investigation and Analysis of Dengue Fever in Xingan, Jiangxi Province from 2018 to 2021 and Research on Preventive Measures. Anti Infect Pharm. 2023;20(02):161-5. doi: 10.13493/j.issn.1672-7878.2023.02-013.

23.	Fan Y, Ya-lan H, Yue L, Xiao-min Z, Ling-hong X, Ren-li Z. Investigation on co － infection status of different serotypes of dengue virus in Shenzhen，2015 －2022. Chin J Health Lab Tec. 2023;33(20):2546-50.

24.	Li-yun J, Wen-zhe S, Wen-hui L, Yi-min C, Biao D, Zhou-bin Z. Epidemiological characteristics of dengue fever and molecular biological

characteristics of envelope gene of dengue virus in Guangzhou, Guangdong, 2015-2019. Disease Surveillance. 2024:1-12.

25.	Ji-jun S, Jie M, Er-ping C, Yu-qi H. Epidemiological and genotypic analysis of 22 imported dengue fever cases in Henan Province. Modern Disease Control and Prevention. 2024;35(09):709-13+17. doi: 10.13515/j.cnki.hnjpm.1006-8414.2024.09.015.

26.	Ting G, Li-ping X, Tao-fa L, You-guang L, Chun-xiang W, Hao L, et al. The epidemiological and clinical

characteristics of 328 patients with dengue fever in Fuzhou. Infect Dis Info. 2024;37(4):364-8. doi: 10.3969/j.issn.1007-8134.2024.04.0014.

27.	Xiao-fang G, Yi-han-yu R, Xing-yun H, Xiang-yin L, Li-yuan C, Xue-fei W, et al. Analysis on etiological surveillance of dengue fever cases at national dengue monitoring sites in Yunnan Province in 2023. China Tropical Medicine. 2025;25(02):141-8. doi: 10.13604/j.cnki.46-1064/r.2025.02.03.

28.	Zheng-hua X. Study on co-infection and clinical features of dengue fever in Guangzhou during 2015 to 2018 and evaluation of enzyme linked immunosorbent assay for thedetection of dengue virus-infected mosquitoes [Master's Thesis]: Southern Medical University; 2019.

29.	Yu H, Kong Q, Wang J, Qiu X, Wen Y, Yu X, et al. Multiple Lineages of Dengue Virus Serotype 2 Cosmopolitan Genotype Caused a Local Dengue Outbreak in Hangzhou, Zhejiang Province, China, in 2017. Sci Rep. 2019;9(1):7345. Epub 20190514. doi: 10.1038/s41598-019-43560-5. PubMed PMID: 31089152; PubMed Central PMCID: PMCPMC6517437.

30.	Jing QL, Yang ZC, Luo L, Xiao XC, Di B, He P, et al. Emergence of dengue virus 4 genotype II in Guangzhou, China, 2010: survey and molecular epidemiology of one community outbreak. BMC Infect Dis. 2012;12:87. Epub 20120412. doi: 10.1186/1471-2334-12-87. PubMed PMID: 22497881; PubMed Central PMCID: PMCPMC3375192.

31.	Xiao JP, He JF, Deng AP, Lin HL, Song T, Peng ZQ, et al. Characterizing a large outbreak of dengue fever in Guangdong Province, China. Infect Dis Poverty. 2016;5:44. Epub 20160503. doi: 10.1186/s40249-016-0131-z. PubMed PMID: 27142081; PubMed Central PMCID: PMCPMC4853873.

32.	Guo C, Lin J, Chen W, Zhang Z, Wu D, Ke C. Molecular Epidemiology of Dengue Viruses Isolated in Shantou City, China, during 2015-2017. Jpn J Infect Dis. 2020;73(4):300-5. Epub 20200228. doi: 10.7883/yoken.JJID.2019.435. PubMed PMID: 32115543.

33.	Lin YP, Luo Y, Chen Y, Lamers MM, Zhou Q, Yang XH, et al. Clinical and epidemiological features of the 2014 large-scale dengue outbreak in Guangzhou city, China. BMC Infect Dis. 2016;16:102. Epub 20160301. doi: 10.1186/s12879-016-1379-4. PubMed PMID: 26932451; PubMed Central PMCID: PMCPMC4774186.

34.	Jiang L, Liu Y, Su W, Liu W, Dong Z, Long Y, et al. Epidemiological and genomic analysis of dengue cases in Guangzhou, China, from 2010 to 2019. Sci Rep. 2023;13(1):2161. Epub 20230207. doi: 10.1038/s41598-023-28453-y. PubMed PMID: 36750601; PubMed Central PMCID: PMCPMC9905598.

35.	Cao J, Deng H, Ye L, Ma X, Chen S, Sun X, et al. Epidemiological and clinical characteristics of Dengue virus outbreaks in two regions of China, 2014 - 2015. PLoS One. 2019;14(3):e0213353. Epub 20190305. doi: 10.1371/journal.pone.0213353. PubMed PMID: 30835769; PubMed Central PMCID: PMCPMC6400443.

36.	Huang J, Zhang M, Li S, Liu J, Lin X, Li Q, et al. Epidemiological and clinical characteristics of dengue fever in Fuzhou, China, in 2023. BMC Infect Dis. 2024;24(1):1275. Epub 20241111. doi: 10.1186/s12879-024-10103-x. PubMed PMID: 39528965; PubMed Central PMCID: PMCPMC11552373.

37.	Yi-quan X, Qing C. Epidemiology of dengue fever in China since 1978. J South Med Univ. 2014;34(12):1822-5.

38.	Dan N, Jiu-feng S, Zhi-qiang P, De W. [Epidemiological Analysis of Dengue Fever in Guangdong Province]. South China J Prev Med. 2017;43(04):368-72. doi: 10.13217/j.scjpm.2017.0368. Chinese.

39.	Ying H, Hai-yun C, Hua H, Jun X, Wen-ming L, Yan W, et al. [Analysis of serotypes and clinical features of 22 dengue fever cases]. JOURNAL OF DALI UNIVERSITY. 2017;2(10):73-8. Chinese.

40.	Jian-hua F, Ting-song H, Hai-lin Z, Jin Z, Hong-bin L, Yan G, et al. Epidemiological investigation on epidemic situation of dengue serotype 1 and 2 viruses in Xishuangbanna Prefecture of Yunnan, 2016. China Tropical Medicine. 2017;17(10):982-7. doi: 10.13604/j.cnki.46-1064/r.2017.10.07.

41.	Zhong-wei C, Li-ping Z, Chang-yong F, Yong H, Yan-fen L, Xin Z. Prevalence and E genes sequences analysis of dengue virus type 1 in Dongguan, 2014-2017. China Tropical Medicine. 2018;18(10):1016-20. doi: 10.13604/j.cnki.46-1064/r.2018.10.10.

42.	Jian-hua F, Yun F, Jin Z, Hong-bin L, Yang G, Wei-ping L, et al. An outbreak of dengue serotype 1 virus in Xishuangbanna prefecture of Yunnan province, China, 2017. DISEASE SURVEILLANCE. 2019;34(05):427-34.

43.	Liang C, Qian⁃lai S, Fang⁃ling H, Hong Z, Shi⁃xiong H, Ge Z, et al. Epidemiology and virological characteristics of the dengue fever surveillance in Hunan province from 2008 to 2016. J Trop Med. 2019;19(11):1425-30+41.

44.	Ling Y, Qi-long T, Bo Y, Ji-yin Y, Lin-fu G, Zhe-en Z, et al. Epidemiological survey of the first outbreak of dengue fever in island area, Zhejiang Daishan county. DISEASE SURVEILLANCE. 2020;35(04):307-10.

45.	Yi-xiong L, Quan-De W, Hui-Tao H, Feng R, Xi-He N. Epidemiological characteristics and E gene evolution of Dengue virus in Zhuhai, 2019. South China J Prev Med. 2021;47(03):359-62.

46.	Jie T, Zhi-hong F, Juan Y, Xue-ying J, Ming-dong Y, Xiao-fang G. Laboratory detection of imported dengue fever cases in Yuxi City, Yunnan Province, 2019. Chinese Journal of Zoonoses. 2021;37(05):416-20.

47.	Ting-ting Y, Qi L, Hong-bin C, Nai-peng K, Yu-wei W, Sheng-gen W, et al. Epidemiological and pathogenic characteristics of dengue fever local outbreaks in Putian City, Fujian Province. Strait J Pre Med. 2022;28(06):5-8+11.

48.	Feng-fu C. The study of characteristics of dengue molecular epidemiology and prevention and control strategies in Guangdong Province [Master Thesis]: Southern Medical University; 2022.

49.	Wei-Ying X, Hang-Jian L, Ji-Ling W. [Epidemiological and clinical characteristics of 10 imported dengue fever cases in Shaoxing, Zhejiang Province]. Chin J Ctrl Endem Dis. 2024;39(05):425-6. Chinese.

50.	Huang L, Luo X, Shao J, Yan H, Qiu Y, Ke P, et al. Epidemiology and characteristics of the dengue outbreak in Guangdong, Southern China, in 2014. Eur J Clin Microbiol Infect Dis. 2016;35(2):269-77. Epub 20151223. doi: 10.1007/s10096-015-2540-5. PubMed PMID: 26700953.

51.	Hu TS, Zhang HL, Feng Y, Fan JH, Tang T, Liu YH, et al. Epidemiological and molecular characteristics of emergent dengue virus in Yunnan Province near the China-Myanmar-Laos border, 2013-2015. BMC Infect Dis. 2017;17(1):331. Epub 20170508. doi: 10.1186/s12879-017-2401-1. PubMed PMID: 28482813; PubMed Central PMCID: PMCPMC5422898.

52.	Sun J, Lu L, Wu H, Yang J, Xu L, Sang S, et al. Epidemiological trends of dengue in mainland China, 2005-2015. Int J Infect Dis. 2017;57:86-91. Epub 20170216. doi: 10.1016/j.ijid.2017.02.007. PubMed PMID: 28214563.

53.	Lai S, Huang Z, Zhou H, Anders KL, Perkins TA, Yin W, et al. The changing epidemiology of dengue in China, 1990-2014: a descriptive analysis of 25 years of nationwide surveillance data. BMC Med. 2015;13:100. Epub 20150428. doi: 10.1186/s12916-015-0336-1. PubMed PMID: 25925417; PubMed Central PMCID: PMCPMC4431043.

54.	Zhang Z, Jing Q, Chen Z, Li T, Jiang L, Li Y, et al. The increasing menace of dengue in Guangzhou, 2001-2016: the most important epicenter in mainland China. BMC Infect Dis. 2019;19(1):1002. Epub 20191127. doi: 10.1186/s12879-019-4504-3. PubMed PMID: 31775646; PubMed Central PMCID: PMCPMC6880554.

55.	Ren J, Chen Z, Ling F, Huang Y, Gong Z, Liu Y, et al. Epidemiology of Indigenous Dengue Cases in Zhejiang Province, Southeast China. Front Public Health. 2022;10:857911. Epub 20220414. doi: 10.3389/fpubh.2022.857911. PubMed PMID: 35493348; PubMed Central PMCID: PMCPMC9046573.

56.	Lun X, Wang Y, Zhao C, Wu H, Zhu C, Ma D, et al. Epidemiological characteristics and temporal-spatial analysis of overseas imported dengue fever cases in outbreak provinces of China, 2005-2019. Infect Dis Poverty. 2022;11(1):12. Epub 20220124. doi: 10.1186/s40249-022-00937-5. PubMed PMID: 35074010; PubMed Central PMCID: PMCPMC8785556.

57.	Jing QL, Cheng Q, Marshall JM, Hu WB, Yang ZC, Lu JH. Imported cases and minimum temperature drive dengue transmission in Guangzhou, China: evidence from ARIMAX model. Epidemiol Infect. 2018;146(10):1226-35. Epub 20180521. doi: 10.1017/s0950268818001176. PubMed PMID: 29781412; PubMed Central PMCID: PMCPMC9134281.
